# Chromatin Dynamics Contribute to the Spatiotemporal Expression Pattern of Virulence Genes in a Fungal Plant Pathogen

**DOI:** 10.1128/mBio.02343-20

**Published:** 2020-10-06

**Authors:** Lukas Meile, Jules Peter, Guido Puccetti, Julien Alassimone, Bruce A. McDonald, Andrea Sánchez-Vallet

**Affiliations:** a Plant Pathology, Institute of Integrative Biology, ETH Zürich, Zürich, Switzerland; b Centro de Biotecnología y Genómica de Plantas (CBGP, UPM-INIA), Universidad Politécnica de Madrid (UPM)—Instituto Nacional de Investigación y Tecnología Agraria y Alimentaria (INIA), Pozuelo de Alarcón (Madrid), Spain; Universidad de Córdoba

**Keywords:** chromatin, effector gene, filamentous fungi, histone methylation, plant pathogens, reporter gene, virulence, wheat

## Abstract

Dynamic changes in transcription profiles are key for the success of pathogens in colonizing their hosts. In many pathogens, genes associated with virulence, such as effector genes, are located in regions of the genome that are rich in transposable elements and heterochromatin. The contribution of chromatin modifications to gene expression in pathogens remains largely unknown. Using a combination of a reporter gene-based approach and chromatin immunoprecipitation, we show that the heterochromatic environment of effector genes in the fungal plant pathogen Zymoseptoria tritici is a key regulator of their specific spatiotemporal expression patterns. Enrichment in trimethylated lysine 27 of histone H3 dictates the repression of effector genes in the absence of the host. Chromatin decondensation during host colonization, featuring a reduction in this repressive modification, indicates a major role for epigenetics in effector gene induction. Our results illustrate that chromatin modifications triggered during host colonization determine the specific expression profile of effector genes at the cellular level and, hence, provide new insights into the regulation of virulence in fungal plant pathogens.

## Abstract (precis)

Fungal plant pathogens possess a large repertoire of genes encoding putative effectors, which are crucial for infection. Many of these genes are expressed at low levels in the absence of the host but are strongly induced at specific stages of the infection. The mechanisms underlying this transcriptional reprogramming remain largely unknown. We investigated the role of the genomic environment and associated chromatin modifications of effector genes in controlling their expression pattern in the fungal wheat pathogen Zymoseptoria tritici. Depending on their genomic location, effector genes are epigenetically repressed in the absence of the host and during the initial stages of infection. Derepression of effector genes occurs mainly during and after penetration of plant leaves and is associated with changes in histone modifications. Our work demonstrates the role of chromatin in shaping the expression of virulence components and, thereby, the interaction between fungal pathogens and their plant hosts.

## INTRODUCTION

The transition of pathogenic fungi from nonhost to host environments requires dynamic changes in gene expression profiles, including the activation of genes with host-specific functions (1–4). Recent work has shown that, in addition to classical transcription factors, chromatin structure contributes to the transcriptional control of genes involved in host colonization (5–8). Genomic regions consisting of loosely packed chromatin (euchromatin) are generally conducive for transcription, while densely packed chromatin (heterochromatin) is less accessible and less easily transcribed (9). A variety of different proteins associated with chromatin and several epigenetic processes, including a complex array of posttranslational histone modifications, work in concert to shape chromatin structure in eukaryotes and thereby provide an important layer of gene regulation (10–13). In euchromatin, lysine residues of histones are frequently acetylated, while hypoacetylated histones are associated with heterochromatin and transcriptionally silent genes (14). Heterochromatin is further characterized by trimethylation of lysine 9 and/or lysine 27 of histone H3 (H3K9me3 and/or H3K27me3, respectively), which are posttranslational modifications catalyzed by the histone methyltransferases KMT1 and KMT6, respectively (12, 15–17). Consequently, derepression of genes residing in heterochromatic regions requires alteration of histone modifications, acting in conjunction with an active transcription machinery (18, 19).

The genomes of filamentous fungi have been described as compartmentalized into euchromatic gene-rich regions containing housekeeping genes and into heterochromatic regions rich in transposable elements (TEs) and poor in genes (17, 20, 21). This compartmentalization has been broadly investigated in plant pathogens and is thought to facilitate different evolutionary rates across the genome (22, 23). Fungal effectors, including small secreted proteins and secondary metabolites, are molecules that have major roles in plant-fungus interactions and enable host and/or niche colonization (24–27). Fungal secondary metabolite gene clusters and genes encoding effector proteins often reside in TE-rich genomic compartments (18, 20, 21, 28–30). This nonrandom distribution of these genes in the genome suggests that TEs might provide fungi with an improved capacity to adapt to their niche or, in the case of plant pathogens, to their host and its immune system (31). TEs are typically associated with repressive epigenetic marks to control their activity. This repression can extend outside the TEs and affect adjacent genes (32–34). Thus, expression of many effector genes can be influenced by their proximity to TEs. For instance, in the oil-seed rape pathogen Leptosphaeria maculans, effector genes are frequently located in TE-rich regions and have been shown to be under epigenetic control involving H3K9me3 (35). Associations of secondary metabolite biosynthetic gene clusters with heterochromatin histone marks have been shown for filamentous fungi such as Aspergillus nidulans, Epichloë festucae, Fusarium fujikuroi, Fusarium graminearum, and Colletotrichum higginsianum (7, 8, 36–38).

Induction of heterochromatic effector and secondary metabolite genes during host colonization in plant-colonizing fungi is thought to require the remodeling of chromatin (6, 18). However, exactly how, when, and where chromatin is reorganized in plant colonizers to induce interaction-specific genes is largely unknown (10, 15). In pioneering work, Chujo and Scott found that in *E. festucae*, secondary metabolite gene upregulation during host colonization was associated with a decrease in H3K27me3 and H3K9me3 levels (7), highlighting that chromatin remodeling is likely critical for shaping the expression pattern of genes involved in host interaction.

**FIG 1:**
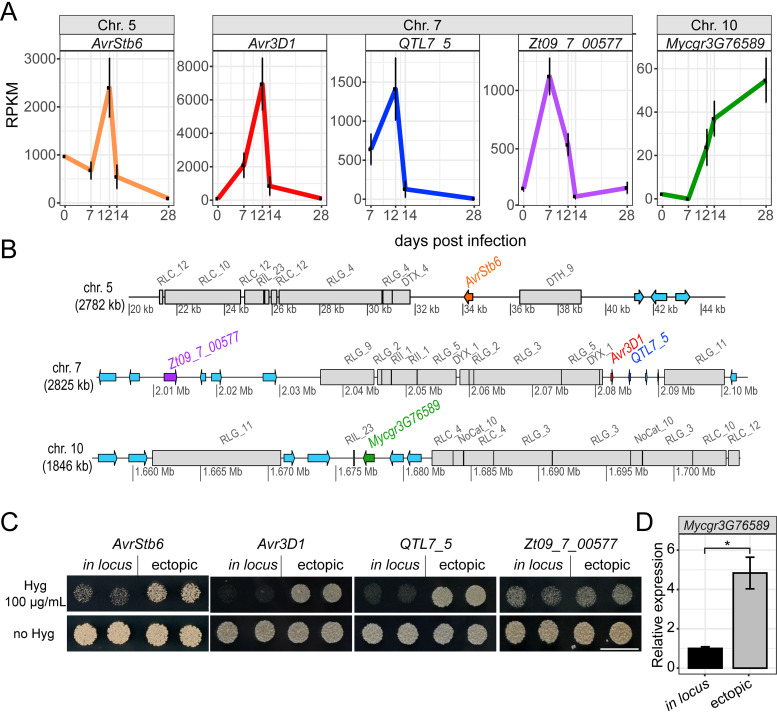
The genomic environment of selected Zymoseptoria tritici effector genes is repressive for expression. (A) Effector genes are induced during host colonization. Data represent expression values of *Zt09_7_00577* and of the effector genes *AvrStb6*, *Avr3D1*, *QTL7_5*, and *Mycgr3G76589* during host colonization. Reads per kilobase per million (RPKM) values were obtained from Palma-Guerrero et al. (48) and Francisco et al. (67). (B) The effector genes *AvrStb6*, *Avr3D1*, *QTL7_5*, and *Mycgr3G76589* reside in transposable element (TE)-rich regions of the genome of strain 3D7. Colored arrows represent genes from their start to their stop codons, and gray blocks represent TEs. Gene and TE annotations are based on work reported previously by Plissonneau et al. (81). TEs were classified according to Wicker et al. (93) as follows: the first letter indicates the class (R = RNA class; D = DNA class); the second letter indicates the order (L = LTR; I = Line; T = TIR; Y = Crypton); and the third letter indicates the superfamily (C = Copia; G = Gypsy; L = L1; I = I; H = PIF-Harbinger; M = Mutator; X = unknown; NoCat = no category). chr.= chromosome. (C) Hygromycin B sensitivity assay with 3D7-derived transformants carrying the hygromycin B resistance gene *Hph* under the control of a constitutive promoter in locus *AvrStb6*, *Avr3D1*, or *QTL7_5* (*in locus*) or at random positions of the genome (ectopic). *Zt09_7 _00577* is a noneffector control locus located ∼70 kb upstream of *Avr3D1*. For both *in locus* and ectopic transformants, two independent lines are shown. Pictures were taken after 6 days of growth at 18°C on YMS agar. The white bar represents 10 mm. Hyg = hygromycin B. (D) Relative expression levels of the effector gene *Mycgr3G76589* inserted ectopically compared to the endogenous gene (*in locus*) in strain 3D7 under axenic conditions (YPD liquid medium). *Actin* was used as a reference gene. *n_in_locus_* = 3, *n*_ectopic_ = 6. Bars represent the means, and error bars represent standard errors of the means. Asterisks represent statistical differences (*P* < 0.05, Student's *t* test).

The wheat pathogen Zymoseptoria tritici (formerly Mycosphaerella graminicola) is an additional example of a plant-pathogenic fungus in which several putative effector genes (28%) and secondary metabolite gene clusters (50%) are associated with repressive histone modifications (H3K27me3 and H3K9me3) (39, 40). These heterochromatin marks influence genome stability in *Z. tritici*, but their roles in gene expression regulation remain enigmatic, since their removal leads to the induction of only a small fraction of genes in the absence of the host (41). However, the location of effector genes in heterochromatic regions raises the possibility that a large fraction of these genes are epigenetically controlled (40). *Z. tritici* is a devastating pathogen that causes necrosis on wheat leaves after an asymptomatic period that lasts more than 7 days (42–44). During this asymptomatic phase, hyphae on the leaf surface penetrate the stomata and colonize the apoplastic space. Necrotic lesions eventually appear simultaneously with the formation of asexual reproductive structures (44–46). The different stages of infection presumably involve different subsets of effectors (47). Consequently, putative effector genes have a distinct expression pattern with very low transcript levels under axenic conditions and high induction at various stages of host colonization (47–49). For example, a gene encoding a predicted effector with a cellulase domain (*Mycgr3G76589*), which was suggested to be an inducer of the immune response, is specifically expressed during the necrotrophic and saprotrophic phases (50) (Fig. 1A). On the other hand, two validated effector genes (*AvrStb6* and *Avr3D1*) and a predicted one (*QTL7_5*) without any known functional domain are expressed at low levels at early stages of the infection and reach maximum expression levels at the onset of the necrotrophic phase, but are not expressed in the saprotrophic phase (51, 52) (Fig. 1A). Understanding how the tight regulation of effector gene expression is achieved remains a fundamental question in plant pathology.

In an effort to determine the contribution of epigenetic changes to the tight control of effector genes, we engineered the *Z. tritici* genome with reporter genes that allowed us to distinguish the contributions of the promoter and the genomic environment to effector gene expression. Our data demonstrate that the repressive genomic environment of effector genes shapes their spatiotemporal expression pattern. We additionally showed that derepression of effector loci requires the activity of promoters that are strongly activated *in planta* and is associated with dynamic chromatin modifications, featuring a reduction of H3K27me3 and, in most of the cases, H3K9me3 levels.

## RESULTS

### The genomic environment has a repressive effect on effector gene expression in the absence of the host.

To test whether the genomic location contributes to the typically low expression levels of effector genes in the absence of the host, we selected the genes *AvrStb6*, *Avr3D1*, *QTL7_5*, and *Mycgr3G76589* for functional analyses. We chose these genes because of their stage-specific expression patterns during host colonization (Fig. 1A) (50–53) and because they were previously shown to be located in heterochromatic regions under axenic conditions in reference strain IPO323 and in nonsyntenic and TE-rich regions of the genomes of strains ST99CH_3D7 (abbreviated here as 3D7) and IPO323 (39, 51, 52) (Fig. 1B; see also Table S1 in the supplemental material). For the effector loci *AvrStb6*, *Avr3D1*, and *QTL7_5*, we inserted different reporter genes either in the loci of interest or ectopically, i.e., in random positions in the genome of *Z. tritici* strain 3D7. Insertion of a hygromycin resistance gene (*Hph*) cassette with a constitutive promoter in the loci *AvrStb6*, *Avr3D1*, and *QTL7_5* resulted in higher sensitivity to hygromycin B of the recipient strains than was seen with ectopic integration of the same cassette in all the tested independent transformant lines (Fig. 1C), suggesting a repressive role of the genomic environment in gene expression at these effector loci. For the noneffector locus *Zt09_7_00577* (53) upstream of *Avr3D1* (Fig. 1B), a repressive role of the genomic environment in expression of the inserted reporter gene was not observed (Fig. 1C). For *Avr3D1* and *QTL7*_5, the same experiment was performed in a different strain, ST99CH_3D1 (abbreviated here as 3D1), with similar results (see Fig. S1A in the supplemental material). Using quantitative reverse transcription-PCR (qRT-PCR), we confirmed that *in locus* insertion of the *Hph* cassette resulted in lower *Hph* transcript levels than were seen with ectopic insertions (Fig. S1B). To further characterize repression of *Avr3D1* and *AvrStb6*, an enhanced green fluorescent protein (eGFP) gene reporter cassette with a constitutive promoter was inserted in these loci. The eGFP fluorescence was lower in *in locus* transformants than in ectopic transformants in both cases and lower for locus *Avr3D1* than for *AvrStb6* (Fig. S1C), demonstrating that repression at the *Avr3D1* and *AvrStb6* loci is independent of the reporter gene and that different loci can be subjected to different levels of repression.

10.1128/mBio.02343-20.1 FIG S1 The genomic environment of selected Zymoseptoria tritici effector genes is repressive for expression. (A) (Top) Construct design for insertion of the *Hph* gene encoding the hygromycin B phosphotransferase used as a reporter for the epigenetic state in the loci *Avr3D1* (left) and *QTL7_5* (right). (Bottom) Hygromycin B sensitivity assay with mutants in the background of strain 3D1 carrying the hygromycin B resistance gene *Hph* either in one of the loci *Avr3D1* and *QTL7_5* (*in locus*) or in random positions of the genome (ectopic). Two independent transformant lines are shown. (B) Relative expression levels of the *Hph* gene inserted ectopically compared to the same gene inserted at locus *Avr3D1* (*in locus*) in strain 3D1 under axenic conditions (YSB medium). The numbers of independent transformant lines were *n_in_locus_* = 4 and *n*_ectopic_ = 2. The 18S gene was used as a reference, and relative expression levels were calculated without efficiency correction. Bars represent the means, and error bars represent the standard errors of the means. Asterisks represent statistical differences (*P* < 0.05, Student’s *t* test). (C) (Top) Construct design for insertion of the *eGFP* reporter cassette in the loci *AvrStb6* (left) and *Avr3D1* (right) to assess the influence of the genomic context on gene expression. (Bottom) eGFP and mCherry fluorescence (Fire lookup table [LUT] of ImageJ software) in transformants harboring the *eGFP* cassette either in locus *AvrStb6* or *Avr3D1* (*in locus*) or in a random position of the genome (ectopic). The transformants were obtained in a 3D7-derived strain carrying the *mCherry* reporter gene at a euchromatic region under the control of the same promoter as the *eGFP* reporter gene (*α-tubulin* promoter, P*α-tub*). T*tef1* = Aspergillus nidulans *tef1* terminator; T*α-tub* = *Z. tritici α-tubulin* terminator; T*trpC* = A. nidulans *trpC* terminator; P*trpC* = A. nidulans *trpC* promoter; *Hph* = hygromycin phosphotransferase gene; chr. = chromosome. The white scale bar represents 20 μm. Download FIG S1, JPG file, 2.3 MB. Copyright © 2020 Meile et al. 2020 Meile et al. This content is distributed under the terms of the Creative Commons Attribution 4.0 International license.

10.1128/mBio.02343-20.6 TABLE S1 Genomic location and chromatin context of the investigated genes under axenic conditions. The genomic location in the genomes of IPO323 and 3D7 is indicated from start to stop codon. n.d. = not determined. Download Table S1, PDF file, 0.1 MB. Copyright © 2020 Meile et al. 2020 Meile et al. This content is distributed under the terms of the Creative Commons Attribution 4.0 International license.

For the effector gene *Mycgr3G76589*, we were unable to obtain *in locus* transformants; we therefore used a different approach to study the effect of the genomic environment on gene expression. We generated transformant lines with a second, ectopically inserted copy of *Mycgr3G76589* under the control of the native promoter and compared the level of expression to that seen with the wild type using qRT-PCR. Expression levels from ectopic sites in six independent mutants were higher than the levels from the native locus (Fig. 1D), indicating that the *Mycgr3G76589* locus is also epigenetically repressed in axenic culture.

**FIG 2:**
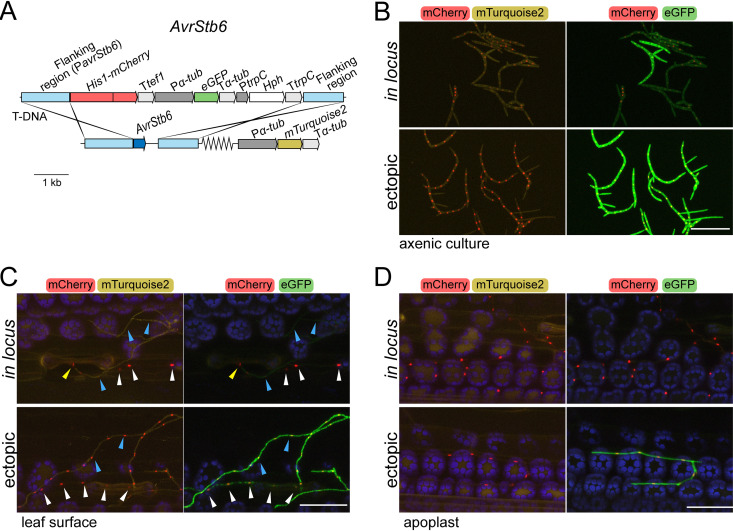
*AvrStb6* is silenced under axenic conditions but derepressed during host colonization. (A) Construct design for the insertion of two reporter genes into the *AvrStb6* locus in Zymoseptoria tritici. It encodes (i) a His1-mCherry fusion protein under the control of the *AvrStb6* promoter (P*avrStb6*, located in the left flanking region) and (ii) eGFP under the control of the constitutive *α-tubulin* promoter (P*α-tub*). The flanking regions consisted of at least 1.173 kb of sequence identical to the 3D7 genome for homologous recombination. T*tef1* = Aspergillus nidulans *tef1* terminator; T*α-tub* = *Z. tritici α-tubulin* terminator; T*trpC* = A. nidulans *trpC* terminator; P*trpC* = A. nidulans *trpC* promoter; *Hph* = hygromycin phosphotransferase gene. Serrated lines indicate different chromosomal locations. To be able to visualize all fungal cells, the strain 3D7 expressing *mTurquoise2* under the control of the *α-tubulin* promoter was used for transformation. (B to D) Fluorescence of mCherry, mTurquoise2, and eGFP of *Z. tritici* strain 3D7 transformed with the construct shown in panel A under axenic conditions (B), during the early stages of infection of wheat leaves (C), and during colonization of the apoplast (D). The construct was inserted in the *AvrStb6* locus (*in locus*) or in random positions of the genome (ectopic). The images on the left show the overlay of the chloroplast autofluorescence (in blue), mCherry (in red), and mTurquoise2 (in yellow) channels, and the images on the right show the overlay of the chloroplast autofluorescence (in blue), mCherry (in red), and eGFP (in green) channels. Maximum intensity z-projections of the confocal images are shown. In panel C, blue arrowheads mark nuclei from cells located on the leaf surface, white arrowheads mark hyphae located in the apoplast, and yellow arrowheads mark nuclei from a cell in contact with a stomate. The images in panel D show hyphae and nuclei located in the apoplast. Scale bars represent 50 μm.

**FIG 3:**
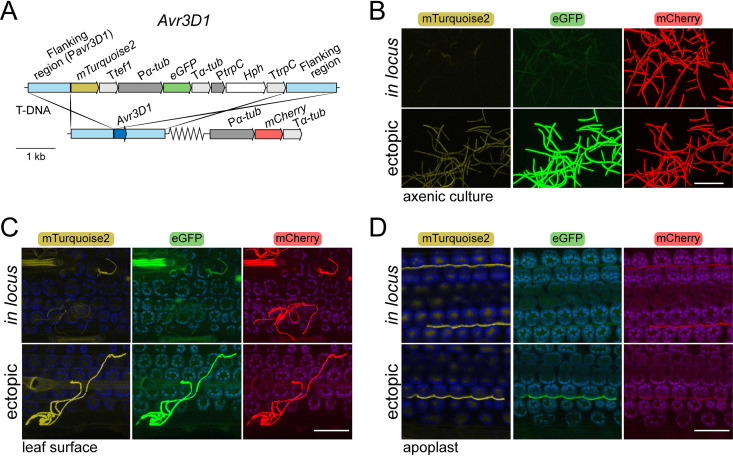
*Avr3D1* is silenced under axenic conditions but derepressed during host colonization. (A) Construct design for the insertion of two reporter genes into the *Avr3D1* locus in Zymoseptoria tritici. The genes encode (i) mTurquoise2 under the control of the *Avr3D1* promoter (P*avr3D1*, located in the left flanking region) and (ii) eGFP under the control of the constitutive *α-tubulin* promoter (P*α-tub*). The flanking regions consisted of at least 1.090 kb of sequence identical to the 3D7 genome for homologous recombination. T*tef1* = Aspergillus nidulans *tef1* terminator; T*α-tub* = *Z. tritici α-tubulin* terminator; T*trpC* = A. nidulans *trpC* terminator; P*trpC* = A. nidulans *trpC* promoter; *Hph* = hygromycin phosphotransferase gene. Serrated lines indicate different chromosomal locations. To be able to visualize all fungal cells, strain 3D7 expressing *mCherry* under the control of the *α-tubulin* promoter was used for transformation. (B to D) Fluorescence of mTurquoise2, eGFP, and mCherry of *Z. tritici* strain 3D7 transformed with the construct shown in panel A under axenic conditions (B), during epiphytic growth on wheat leaves (C), and during colonization of the apoplast (D). The construct was inserted either in the *Avr3D1* locus (*in locus*) or in random positions of the genome (ectopic). The images on the left show the overlay of the chloroplast autofluorescence (in blue) and mTurquoise2 (in yellow) channels; the images on the middle show the overlay of chloroplast autofluorescence (in blue) and eGFP (in green) channels; and the images on the right show the overlay of the chloroplast autofluorescence (in blue) and mCherry (in red) channels. Maximum intensity z-projections of the confocal images are shown. Scale bars represent 50 μm.

We further tested whether *in locus*-inserted fluorescent reporter genes were also repressed when they were under the control of the native promoters of *AvrStb6* (P*avrStb6*) and *Avr3D1* (P*avr3D1*). To be able to visualize fungal cells, a 3D7-derived recipient strain expressing *mTurquiose2* under the control of a constitutive promoter was used. P*avrStb6* was used to control *mCherry* fused to *His1* to localize the reporter to the nucleus and to thereby monitor the activity of the *AvrStb6* promoter on the single-cell level (Fig. 2A). An additional reporter, *eGFP*, under the control of a constitutive promoter (P*α-tub*) was introduced adjacently to *His1-mCherry* (Fig. 2A) to be able to distinguish the contributions of the promoter and the genomic location to gene expression regulation. In axenic culture, levels of mCherry controlled by the native effector promoter were lower in all tested *in locus* transformants than in all tested ectopic transformants (Fig. 2B), suggesting that *AvrStb6* was probably under epigenetic control in the absence of the host. Additionally, the *eGFP* gene under the control of the constitutive promoter P*α-tub* was repressed when positioned in the effector locus. In a similar way, we also analyzed if the *Avr3D1* locus was silenced. In a 3D7-mCherry strain, P*avr3D1* was used to control *mTurquiose2* expression and P*α-tub* to control *eGFP* expression (Fig. 3A). Similarly to what we observed for the *AvrStb6* locus, the expression of both reporter genes was lower when they were located in the *Avr3D1* locus than when they were placed ectopically (Fig. 3B). The simultaneous repression of both reporter genes oriented in tandem in *in locus* transformants suggests that the mechanisms responsible for silencing at the *Avr3D1* and *AvrStb6* loci are potent enough to act on a scale larger than a single gene.

### Effector genes are derepressed *in planta*, and the derepression pattern is disturbed by ectopic gene relocation.

Since effector genes, including *AvrStb6* and *Avr3D1*, are highly induced during host colonization (Fig. 1A), we hypothesized that they are eventually derepressed in the presence of the host. The fluorescent reporter genes driven by the native promoters provided a tool to study the details of derepression of effector genes on the spatial and temporal levels. Strain 3D7, which was used for targeted insertion of the reporter genes, harbors alleles of the avirulence genes *AvrStb6* and *Avr3D1* that are not recognized by the corresponding resistance proteins (Stb6 for *AvrStb6* and Stb7 or Stb12 for *Avr3D1*), and the loss of these genes therefore does not affect infection (51, 52). During infection of wheat leaves, strains with P*avrStb6-His1-mCherry* placed in the *AvrStb6* locus showed high mCherry levels mostly in cells that grow inside the host leaf and in cells close to penetration sites but not in cells of hyphae growing epiphytically (Fig. 2C). However, when the same construct was inserted ectopically, mCherry fluorescence was more uniform and widely detected in hyphae growing on the leaf surface (Fig. 2C), suggesting that relocation of *AvrStb6* to a new place in the genome causes misregulation during the early stages of host colonization and that contact with the host alone is not sufficient for effector gene derepression in the case of *AvrStb6*. Genomic location-dependent repression was restricted to early infection stages, as no differences in mCherry levels between *in locus* and ectopic transformants could be observed inside the host tissue (Fig. 2C and D). Interestingly, *eGFP* under the control of a constitutive promoter and positioned 1.63 kb downstream of the stop codon of the *mCherry* reporter in the *AvrStb6* locus remained largely silent in *in locus* transformants during infection, even in hyphae that had undergone effector derepression (Fig. 2C and D). Thus, derepression seems to be locally restricted and does not extensively affect neighboring loci in the case of *AvrStb6*; i.e., the mechanisms responsible for repression seem to persist during infection in the case of the normally constitutive *α-tubulin* promoter but not in the case of the *AvrStb6* promoter.

For *Avr3D1*, the derepression pattern observed during infection was similar to that seen with *AvrStb6*; however, compared to P*avrStb6-His1-mCherry*, P*avr3D1-mTurquiose2* was derepressed in some hyphae shortly after spore germination (2 days postinfection [dpi]) on the leaf surface independently of their position relative to stomata (Fig. 3C). As in the case of P*avrStb6-His1-mCherry*, ectopic relocation of P*avr3D1-mTurquiose2* led to early activation of the promoter, since the reporter gene was highly expressed in all observed hyphae already at early stages in epiphytic hyphae (Fig. 3C). Full derepression of the *Avr3D1* locus occurred during apoplast colonization since mTurquiose2 accumulated to similar levels in all the transformants regardless of the location of the reporter gene in the genome (Fig. 3D). Remarkably, during infection, *eGFP* remained mostly repressed despite the use of a constitutive promoter to control its expression and its location adjacent to the derepressed *mTurquoise2* (Fig. 3C and D). In conclusion, the genomic location of effector loci is repressive during early stages of the infection and local derepression is dependent on the promoter of the effector genes.

### Histone modifications are involved in effector gene regulation.

Given the location of *AvrStb6*, *Avr3D1*, *QTL7_5*, and *Mycgr3G76589* in TE-rich regions of the 3D7 genome, the lack of cytosine methylation (54), and the enrichment of these genes in the heterochromatin mark histone H3K9me3 or H3K27me3 or both in the IPO323 reference strain (39–41), we hypothesized that those histone modifications are involved in repression of these effector genes in axenic culture. The strong and durable silencing phenotype of the *eGFP* reporter cassette with a constitutive promoter inserted in the *Avr3D1* locus provided a tool to investigate the mechanistic basis of effector repression. On the basis of the described role of histone acetylation as an important determinant of chromatin structure (12), we tested whether increased histone acetylation levels are sufficient to rescue the repression phenotype of the *eGFP* cassette in the context of the *Avr3D1* locus. Treatment with the histone deacetylase inhibitors suberoylanilide hydroxamic acid (SAHA) and trichostatin A (TSA) led to an induction of the previously silenced *eGFP* (Fig. S2), highlighting the epigenetic nature of repression at the *Avr3D1* locus and suggesting a role of histone hypoacetylation in this process.

10.1128/mBio.02343-20.2 FIG S2 Activation of the *Avr3D1* locus by histone deacetylase inhibitor-mediated hyperacetylation. A histone deacetylase inhibition assay was performed with Zymoseptoria tritici transformants harboring the *eGFP* reporter gene controlled by the constitutive *α-tubulin* promoter either at the *Avr3D1* locus (*eGFP*_inlocus_) or at an ectopic location (*eGFP*_ectopic_). Transformants were grown axenically and treated with histone deacetylase inhibitor suberoylanilide hydroxamic acid (SAHA; A) or trichostatin A (TSA; B). All transformants contained the *mCherry* gene under the control of the *α-tubulin* promoter, allowing visualization of living cells regardless of their eGFP levels. A transformant line harboring only *mCherry* and no *eGFP* is also shown. eGFP and mCherry fluorescence is shown using the Fire lookup table (LUT) of ImageJ software. SAHA-treated cells were grown in liquid minimal medium and observed after 11 days. TSA-treated cells were grown in liquid YSB medium and observed after three days. Scale bars represent 50 μm. Download FIG S2, JPG file, 2.7 MB. Copyright © 2020 Meile et al. 2020 Meile et al. This content is distributed under the terms of the Creative Commons Attribution 4.0 International license.

**FIG 4:**
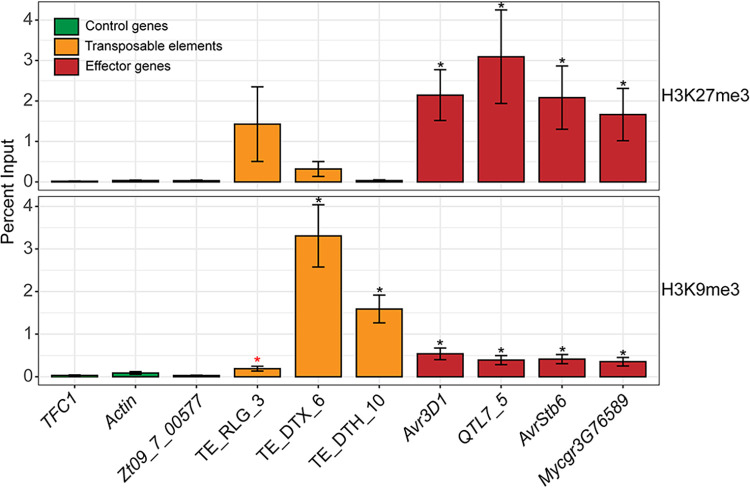
Effector genes are enriched in histone H3 lysine 27 and lysine 9 trimethylation under axenic conditions. Data represent enrichment of histone lysine 27 and lysine 9 trimethylation levels (H3K27me3 and H3K9me3; upper and lower panels, respectively) as measured by the percent input method. Shown are the enrichments for the two housekeeping genes *TFC1* and *Actin* (green bars), the cell wall protein-encoding gene *Zt09_7_00577* residing upstream of the *Avr3D1* effector cluster, three transposable elements (orange bars), and the four effector genes *Avr3D1*, *QTL7_5*, *AvrStb6*, and *Mycgr3G76589* (red bars). Three transposable elements (TEs) with unique sequences in the 3D7 genome were included as controls. The TEs were classified according to Wicker et al. (93): the first letter indicates the class (R = RNA class; D = DNA class); the second letter indicates the order (L = LTR; T = TIR); and the third letter indicates the superfamily (G = Gypsy; H = PIF-Harbinger; X = unknown). Error bars represent standard errors of the means of results from three biological replicates. Black asterisks indicate enrichments that were significantly higher than those seen with *TFC1* and *Actin*; the red asterisk indicates enrichment that was significantly higher than that seen with *TFC1* only (Student's *t* test, *P* < 0.05).

**FIG 5:**
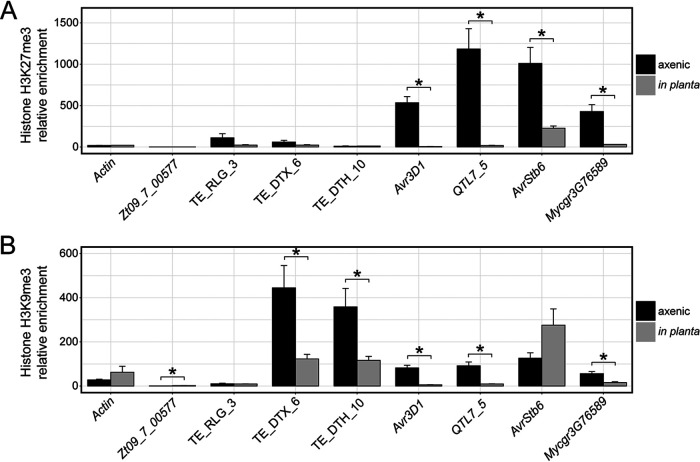
Chromatin is modified in effector loci during host colonization. Data represent relative levels of enrichment of histone H3 lysine 27 trimethylation (A) and histone H3 lysine 9 trimethylation (B) of the four effector genes *Avr3D1*, *QTL7_5*, *AvrStb6*, and *Mycgr3G76589* during axenic growth and during infection at the beginning of the necrotrophic phase. *Actin* is not induced during host colonization, and *Zt09_7_00577* is a noneffector gene located upstream of *Avr3D1*. Three transposable elements (TEs) with unique sequences in the 3D7 genome were included as controls. The TEs were classified according to Wicker et al. (93): the first letter indicates the class (R = RNA class; D = DNA class); the second letter indicates the order (L = LTR; T = TIR); and the third letter indicates the superfamily (G = Gypsy; H = PIF-Harbinger; X = unknown). Error bars represent standard errors of the means of results from three biological replicates. For levels of H3K27me3 in *Actin* and *Zt09_7_00577* and Mycgr3G76589 *in planta*, two replicates were obtained. The relative enrichment data were calculated using a reference housekeeping gene (*TFC1*). Asterisks indicate significant differences between axenic and *in planta* growth (Student's *t* test, *P* < 0.05).

To test whether the four studied effector genes are heterochromatic in the strain 3D7, chromatin immunoprecipitation followed by quantitative PCR (ChIP-qPCR) was performed on axenically grown 3D7 tissue. The enrichment of H3K9me3 and—to a greater extent—H3K27me3 was higher in all four effector genes than in the housekeeping genes *TFC1* (*Mycgr3G110539*), encoding an RNA polymerase III transcription factor subunit, and *Actin* and in the noneffector gene *Zt09_7_00577* located upstream of the *Avr3D1* effector cluster (Fig. 4). In contrast, levels of the euchromatin mark H3K4me2 were lower in the tested effector genes *Avr3D1* and *QTL7_5* than in *TFC1* (Fig. S3). On the basis of the upregulation of the four effector genes *in planta*, we hypothesized that the establishment of the interaction would coincide with a reduction in the level of H3K9me3 or of H3K27me3 or both. We therefore sought to measure H3K9me3 and H3K27me3 levels during host colonization at the onset of the necrotrophic phase, where we expected the four effector genes to reach high expression levels (50, 51, 53) (Fig. 1A). ChIP-qPCR revealed that, during infection, H3K27me3 levels decreased between 4-fold (±1-fold) and 100-fold (±20-fold) for all four tested effector genes and, similarly, that H3K9me3 levels decreased between 4-fold (±1-fold) and 16-fold (±4-fold) for all effector genes except *AvrStb6* (Fig. 5). This reduction in the levels of heterochromatin marks suggests the occurrence of changes in the chromatin structure during host colonization at specific loci, which might contribute to the specific expression pattern of effector genes.

10.1128/mBio.02343-20.3 FIG S3 Effector genes are not enriched in euchromatin mark histone H3 lysine 4 dimethylation. Data represent enrichment of histone lysine 4 dimethylation, lysine 27 trimethylation, and lysine 9 trimethylation levels (H3K4me2, H3K27me3, and H3K9me3; left, middle, and right panels, respectively) under axenic conditions as measured by the percent input method. Chromatin immunoprecipitation was performed as described previously by Soyer et al. (Fungal Genet Biol, 2015, 79:63–70, https://doi.org/10.1016/j.fgb.2015.03.006). Shown are the enrichments for one housekeeping gene (*TFC1*), cell wall protein-encoding gene *Zt09_7_00577* residing upstream of the *Avr3D1* effector cluster, and the two effector genes *Avr3D1* and *QTL7_5*. Error bars represent standard errors of the means of results from three technical replicates. Black asterisks indicate significantly different enrichments compared to *TFC1* (*P* < 0.05, Student’s *t* test). Download FIG S3, JPG file, 0.3 MB. Copyright © 2020 Meile et al. 2020 Meile et al. This content is distributed under the terms of the Creative Commons Attribution 4.0 International license.

**FIG 6:**
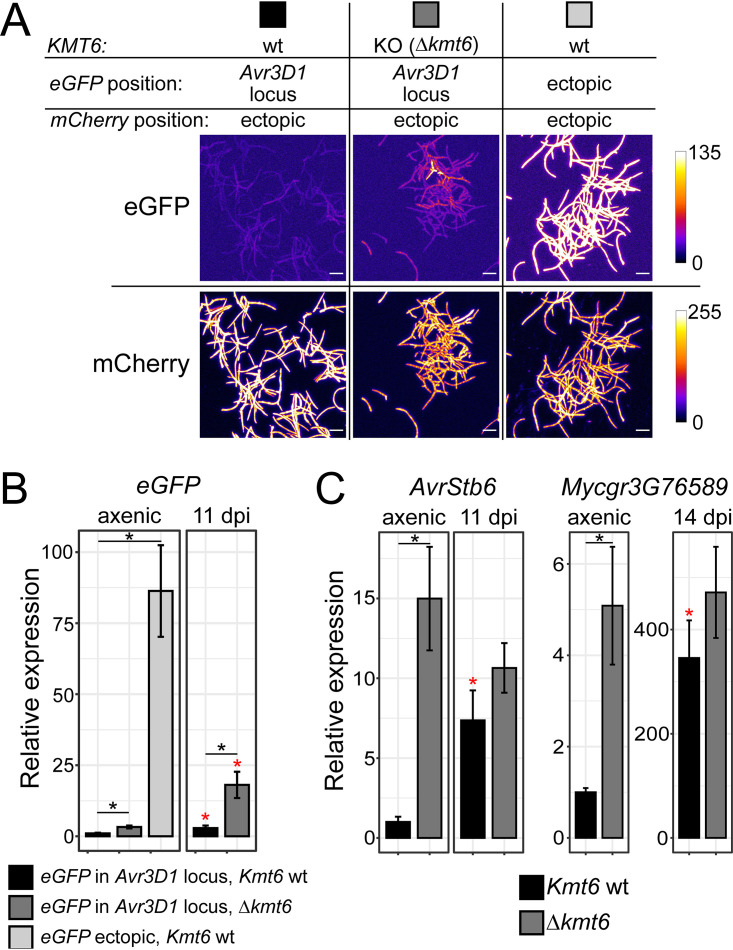
Histone H3 lysine 27 trimethylation is required for effector gene repression. (A) eGFP fluorescence of Zymoseptoria tritici transgenic lines with and without the histone methyltransferase gene *KMT6* (wt and KO, respectively), both harboring the *eGFP* gene under the control of a constitutive promoter in the *Avr3D1* locus. A line harboring an ectopic copy of the *eGFP* cassette is shown as a control. All lines were obtained in a genetic background containing an *mCherry* reporter cassette for visualization of fungal cells. (B) *eGFP* transcript levels in the transgenic lines described in the panel A legend during axenic growth in rich medium and during plant colonization at 11 days postinfection (dpi). (C) Transcript levels of *AvrStb6* and *Mycgr3G76589* during axenic growth in rich medium and during plant colonization in *Z. tritici* lines with and without *KMT6*. Expression levels were normalized to the line with the wild-type *KMT6* gene during axenic growth. *Actin* was used as reference gene for qRT-PCR. Error bars represent standard errors of the means. Data from at least three independent replicates are shown. Black asterisks indicate differences between strains with and without *KMT6*, and red asterisks indicate differences between axenic and *in planta* growth of the same mutant line (*P* < 0.05, Student's *t* test). wt = wild type; KO = knockout.

Considering the dynamic H3K27me3 levels in all four effector genes, we further investigated the role of this histone modification in effector regulation. We obtained a knockout mutant in *KMT6*. *KMT6* was previously demonstrated to be the gene encoding the only histone methyltransferase responsible for H3K27 trimethylation in *Z. tritici*, and it was also shown that loss of H3K27me3 does not affect the distribution of H3K9me3 (41). The *Δkmt6* knockout mutant was obtained in the background of a strain harboring a repressed *eGFP* reporter cassette at the *Avr3D1* locus under the control of a constitutive promoter (Fig. S1C, right panel). The levels of virulence and asexual reproduction of two independent *Δkmt6* mutants were comparable to the control results (Fig. S4B and C); however, we noticed increased adhesion of *Δkmt6* blastospores to agar-based media. In accordance with a possible role of KMT6 in gene repression, *Δkmt6* lines exhibited higher levels of eGFP under axenic conditions (Fig. 6A and B; see also Fig. S4A); however, this level was still substantially lower than in lines with an ectopic insertion of the *eGFP* cassette (Fig. 6A and B), suggesting a small contribution of KMT6 to repression of the *Avr3D1* locus in the absence of the host. Consistent with the low eGFP fluorescence observed *in planta* (Fig. 3C and D), *eGFP* transcript levels measured by RT-qPCR increased only slightly during infection in *in locus* transformant lines (Fig. 6B). However, loss of KMT6 further contributed to increased *eGFP* expression *in planta*, indicating that colonization can trigger partial derepression of a silenced but otherwise constitutive promoter located in a heterochromatic region. As expected, the expression level of the noneffector gene *Zt09_7_00577* was not altered upon removal of *KMT6* (Fig. S4D). The transcript levels of *AvrStb6* and *Mycgr3G76589* were higher in the *Δkmt6* line than in the untransformed control in axenic culture (Fig. 6C). However, this difference was lost during infection (Fig. 6C), suggesting that KMT6 has a repressive effect on these effector genes in the absence of the host and that repression is lost at infection stages of high effector gene expression, which is in line with the reduced H3K27me3 levels observed *in planta*.

10.1128/mBio.02343-20.4 FIG S4 Histone H3 lysine 27 trimethylation is required for effector gene repression. (A) eGFP fluorescence of two independent Zymoseptoria tritici transgenic lines without the histone methyltransferase gene *KMT6* (*Δkmt6*) and three independent transformation controls (*KMT6* wild type), both harboring the *eGFP* gene under the control of a constitutive promoter in the *Avr3D1* locus. All lines were obtained in a genetic background containing an *mCherry* reporter cassette for visualization of fungal cells. eGFP and mCherry fluorescence is displayed using the Fire LUT of ImageJ. (B) Virulence phenotype, measured as the percentage of leaf area covered by lesions, at 17 and 20 days postinfection (dpi) of the *Δkmt6* mutant. The left and right panels show independent experiments. (C) Asexual reproduction, estimated as pycnidium density (pycnidia per cm^2^ lesion) at 17 and 20 dpi of the *Δkmt6* mutant. The left and right panels show results of independent experiments. Between 9 and 16 leaves were analyzed per line. No significant differences were observed between the knockout lines and the control (α = 0.05, Kolmogorov-Smirnov test). n.d. = not determined. (D) The expression of *Zt09_7_00577* is not altered in the *Δkmt6* mutant. Transcript levels of *Zt09_7_00577* during axenic growth in rich medium and during plant colonization (11 dpi) in *Z. tritici* lines with and without *KMT6* were determined. Expression levels were normalized to the line with the wild-type *KMT6* gene during axenic growth. *Actin* was used as the reference gene for qRT-PCR. Error bars represent standard errors of the means. Data from at least three independent replicates are shown. No statistical differences between strains with and without *KMT6* were detected in axenic and *in planta* growth (*P* < 0.05, Student’s *t* test). wt = wild type; KO = knockout. Download FIG S4, JPG file, 1.1 MB. Copyright © 2020 Meile et al. 2020 Meile et al. This content is distributed under the terms of the Creative Commons Attribution 4.0 International license.

## DISCUSSION

A hallmark of fungal and oomycete effector genes is their plant-associated upregulation. Typically, effectors are highly induced during specific stages of host colonization, presumably in accordance with their function during these stages (47). However, knowledge on the molecular mechanisms governing this tight regulation remains scarce, especially with respect to the role of epigenetics in this process. In this work, we show that the heterochromatic environment of effector genes is crucial for controlling their specific expression in a temporal and spatial manner and thereby provides an important layer of regulation. We propose that effector gene derepression during host colonization is locally confined to specific loci and associated with dynamic chromatin modifications, featuring a reduction of H3K27 and/or H3K9 trimethylation levels.

### How are effector genes silenced?

The four effector genes *AvrStb6*, *Avr3D1*, *QTL7_5*, and *Mycgr3G76589* are silenced under axenic conditions. However, their native promoter sequences were not sufficient to induce repression; instead, the broader genomic context influenced their chromatin state and, consequently, their expression profile. Genes involved in host interaction often reside in TE-rich regions in plant-colonizing organisms (6, 31, 39, 40, 55) and have been shown to be under epigenetic control in plant colonizers such as F. graminearum (8), *L. maculans* (35), and *E. festucae* (7). In *Z. tritici*, effector genes induced at the necrotrophic phase are preferentially located in the proximity of TEs and are enriched in H3K27me3 under axenic conditions (40). TEs are usually silenced as a genome defense mechanism (56, 57), and it is thought that TE-associated repressive chromatin modifications are not locally confined but spread to nearby regions, leading to repression of genes therein (32, 58, 59). A repressive role of TEs in nearby genes has been shown for the basidiomycete Pleurotus ostreatus and other fungal species, including the plant symbionts Laccaria bicolor and F. graminearum (60). In *Z. tritici*, TE-mediated repression of proximal genes was demonstrated by deleting a TE cluster, which resulted in derepression of a secondary metabolite gene cluster located 1.9 kb downstream (33). The effector genes investigated in this work reside within similar distances with respect to upstream TE insertions (distance to start codon of between 1.3 and 5.4 kb) and could therefore be subjected to similar repressive influences. However, the TE insertion present 1.3 kb upstream of *Avr3D1* in strain 3D7 is absent in strain 3D1 (51). This TE presence/absence polymorphism does not seem to impact the position effect observed at this locus, since the *Hph* gene inserted in *Avr3D1* was silenced in both strains. Interestingly, *Avr3D1* resides in a genomic region that probably originated from an accessory chromosome. This region is part of the right arm of chromosome 7 and is characterized by high H3K27me3 enrichment and low transcription (39, 47), which are typical features of accessory chromosomes (39). However, the GC content is similar to that in the rest of chromosome 7 and, even in large (up to 210 kb) segments without TEs inside this peculiar region, H3K27me3 enrichment is uniformly high in reference strain IPO323 (39), suggesting that additional features of this region are critical for chromatin architecture.

*AvrStb6* and *Mycgr3G76589* are located in the proximity of the telomeres, which might also influence their chromatin state. Indeed, telomeric repeats are sufficient for widespread H3K27me3-mediated heterochromatin formation in adjacent regions in Neurospora crassa, contributing to their epigenetic control (61). Consequently, subtelomeric regions are frequently heterochromatic (18, 39) and likely influence the expression of genes therein.

By disrupting the gene encoding the histone methyltransferase KMT6, we showed that H3K27me3 had a repressive effect on the investigated effector loci in the absence of the host. However, we observed that for the *Avr3D1* locus, KMT6 made only a minor contribution to repression, since the reporter remained partially silenced in the disruption mutant, suggesting a major role of modifications of histones other than H3K27me3 or other chromatin components, at least for *Avr3D1*. Additional mechanisms involved in transcriptional regulation of genes in heterochromatic regions were also suggested by Möller and colleagues, since they showed that in a different strain of *Z. tritici*, loss of KMT6 under axenic conditions led to an induction of only a small fraction of genes (41). Since disruption of *KMT1* also led to higher expression of only a small amount of genes, heterochromatic marks other than H3K9 and H3K27 trimethylation were suggested to be involved in gene repression under axenic conditions (41). Interestingly, all the investigated effector genes exhibited high levels of both heterochromatin marks in the absence of the host and a reduction of H3K27me3 levels during infection. Similarly, except for *AvrStb6*, H3K9me3 levels also decreased during host colonization, suggesting that these modifications are possibly involved in repression of effector genes and that removal of these marks might enable induction of the genes *in planta*. Not only depletion of H3K27me3 but also inhibition of histone deacetylases led to derepression of the *Avr3D1* locus, indicating that hypoacetylation could also contribute to the silencing of effector genes. In fact, several histone deacetylases are known to target silencing to specific regions of the genome (62, 63); similar mechanisms might operate to repress expression of effector genes in the absence of the host. Chromatin structure and dynamics are complex, involving more than 400 known histone posttranslational modifications (64) and potentially thousands of interacting proteins (13). Thus, it is not surprising that two or more histone modifications contribute to the regulation of a specific locus. In line with the high complexity of chromatin architecture and function, the H3K9me3 enrichment pattern of *AvrStb6* was distinct from the patterns seen with the rest of the investigated effector genes; thus, different regulatory mechanisms might operate at different effector loci.

### How are effector genes derepressed?

We have shown that despite the silenced state of different effector genes in the absence of the host, their derepression is rapidly induced at specific stages of the infection and, in the case of *AvrStb6*, even preferentially in cells that encounter stomata. The *in planta* derepression was associated with a reduction in H3K27me3 and/or H3K9me3 levels, similarly to what was observed for secondary metabolite gene clusters in *E. festucae* (7). Interestingly, during host colonization, H3K9me3 levels in the *AvrStb6* locus remained high despite high levels of expression during this stage, suggesting that removal of this H3K9me3 mark is not always necessary for derepression. The specific derepression of effector genes *in planta* suggests an environmental or developmental trigger, which remains unknown for the *Z. tritici*-wheat interaction. Interestingly, *Avr3D1* and an additional 16 (7%) of 238 (65) candidate effector genes predicted by EffectorP (66) are co-upregulated in four strains during infection and hyphal growth in axenic culture (67). The regulation of this subset of effector genes might be therefore linked to the dimorphic switch, which involves the transcription factor Zt10320 (68). However, most of the hyphal-growth-induced effector genes, including *Avr3D1*, are upregulated *in planta* compared to axenic hyphal growth (67) and our derepression assays showed that *Avr3D1* and *AvrStb6* are still largely repressed at early stages in most individuals on the leaf surface despite growing as hyphae. Therefore, to derepress effector genes, dimorphic switching alone is not sufficient and host colonization is also required.

Previously, it was demonstrated in *L. maculans* that the genomic location strongly influences the expression pattern of effector genes under axenic conditions (35). Similarly, we showed that effector gene location in *Z. tritici* is critical for their low expression levels in the absence of the host. The spatiotemporal derepression patterns of effector genes and the associated local changes in the chromatin state were investigated in this work using fluorescent reporters that informed us about the accessibility of effector loci for the transcription machinery during plant infection. The promoter sequences of *Avr3D1* and *AvrStb6* but not a constitutive promoter were sufficient for *in planta* derepression of a reporter construct inserted in both loci. On the basis of these results, we suggest that sequence-specific DNA-binding proteins contribute to chromatin decondensation at these loci. Transcription factors have been shown to recruit other factors that promote changes in the chromatin structure and thereby regulate transcription (69). For example, the transcription factor PfSIP2 from Plasmodium falciparum binds to a conserved recognition sequence in heterochromatic domains and promotes silencing of virulence genes (70). Interestingly, the relatively small distance (1.6 kb between stop codon and start codon) between the genes that were derepressed *in planta* and the downstream gene that largely remained silent at the same time suggests that only a few nucleosomes are affected by locus-specific chromatin modifications and that specific DNA-binding factors are required for derepression at the locus level. In line with this hypothesis, the level of expression of the closest retrotransposon upstream of *Avr3D1* in strain 3D7 remained low (55) despite the high level of induction of *Avr3D1*. The presence of locally confined chromatin modifications could reflect the necessity to avoid broad-scale derepression of TE-rich regions, reducing deleterious effects associated with TE activation.

### Is an epigenetic layer of regulation needed?

Although effectors generally play a major role in pathogenicity, misexpression may have fatal consequences for the pathogen for various reasons. First, effectors may trigger host defense responses through direct or indirect recognition by host resistance proteins, as shown for both AvrStb6 and Avr3D1. Tight regulation of these avirulence effectors could limit the negative effects of the host immune responses, which may be especially relevant for avirulence factors such as Avr3D1 that induce only partial resistance (51). The location of effector genes in heterochromatic regions might also make them more prone to epiallelic variation, which provides a reversible mechanism to escape avirulence effector recognition (71, 72, 94). Second, in hemibiotrophic pathogens that require living host tissue during colonization, effectors needed for the transition to the necrotrophic infection stage might induce early necrosis if they are expressed prematurely. Some necrotrophic effectors may also induce host defense responses through their functions; for example, secreted cell wall degrading enzymes produce degradation products that can trigger defense responses (73, 74) that might have fatal consequences due to early induction of host defenses. Third, some effectors function as toxins. Although many necrotrophic effectors have plant-specific targets (75), others act as nonspecific toxins (76, 77), and it is possible that some of them exhibit autotoxicity that could be reduced by tight regulation. Fourth, given the typically high expression levels of effector genes during infection (1, 47, 78, 79), the possibility cannot be excluded that an extra layer of regulation would reduce the metabolic costs associated with leaky expression at stages when effectors are not needed.

We hypothesize that the epigenetic layer of gene regulation observed in our experiments provides a key element for regulation of effector genes, contributing to transcriptional inactivity when not needed and thereby reducing the consequences of the host defenses induced upon effector perception and the self-damage caused by secreted enzymes or toxins. Epigenetic mechanisms may also enable stage-specific gene induction that can operate together with or as an alternative to classical transcriptional activators and repressors. Our experiments performed on the *AvrStb6* and *Avr3D1* loci showed that derepression is highly local and likely does not occur without sequence-specific factors as well as one or more host-related triggers, both of which remain to be discovered.

## MATERIALS AND METHODS

### Fungal and bacterial strains, culture conditions, and genome resources.

The Swiss Zymoseptoria tritici strains ST99CH_3D7 and ST99CH_3D1 (80) (abbreviated as 3D7 and 3D1, respectively) and mutants in these backgrounds were used in this study. To assess the proximity of selected effector genes and transposable elements (TEs), we used the 3D7 genome assembly and TE annotations that were previously published (81). For all experiments involving Δ*kmt6* mutants, blastospores were grown on yeast extract-peptone-dextrose agar (YPD agar; 1% yeast extract, 2% peptone, 2% dextrose, 1.5% agar). For all other experiments, either yeast extract-sucrose broth (YSB; 1% yeast extract, 1% sucrose) or yeast extract-malt-sucrose agar (YMS agar; 0.4% yeast extract, 0.4% malt extract, 0.4% sucrose, 1.5% agar) was used if not stated otherwise. For all axenic cultures of *Z. tritici*, media were supplemented with kanamycin sulfate (50 μg/ml). Molecular cloning and plasmid propagation were performed with Escherichia coli strain HST08 (TaKaRa Bio Inc., Shiga, Japan). For Agrobacterium tumefaciens-mediated transformation of *Z. tritici*, the A. tumefaciens AGL-1 strain was used.

### Generation of *Z. tritici* mutant lines.

All constructs for targeted or ectopic insertion mutagenesis were generated with an In-Fusion HD cloning kit (TaKaRa Bio Inc., Shiga, Japan) as previously described (51). All constructs, fragments from which they were assembled, and transformant lines generated in this study are listed in Table S2 in the supplemental material, and the primer sequences are listed in Table S3. Constitutive promoters, terminators, selection markers, and fluorescent reporter genes were amplified from plasmids pES1 and pES6 (plasmids for fungal transformation; E. H. Stukenbrock, Christian-Albrechts-University of Kiel, unpublished data), pFC332 (82), pCGEN (83), pCmCherry (84), and pCZtGFP (95). The fluorescent reporter gene *mTurquoise2* was designed based on the codon-optimized *eGFP* sequence present in pCZtGFP (95), and its double-stranded DNA sequence (see Data Set S1 in the supplemental material) was purchased from IDT (Coralville, IA, USA). His1 was amplified from 3D7 DNA, according to Kilaru et al. (96). *Z. tritici* was transformed by Agrobacterium tumefaciens-mediated transformation as described before (51, 86). Strain 3D7 harboring the *mCherry* gene under the control of the *Z. tritici α-tubulin* promoter was obtained by S. Kilaru and G. Steinberg using targeted ectopic integration (85). For targeted insertion mutants, the insertion was positioned after the promoter in all cases and the position of the insertion was verified by PCR using a primer specific for the insertion sequence combined with a primer specific for the genomic region adjacent to the point of insertion. For all mutants, the copy number of the inserted transgenes was determined by qPCR performed on genomic DNA using the primers listed in Table S3; transformant lines with two or more insertion copies were excluded from further experiments. In the case of Δ*kmt6* mutants, we managed to obtain only two targeted insertion mutants. Although one of them (line 2) had an additional copy of the insertion, we used it as an independent transformant.

10.1128/mBio.02343-20.7 TABLE S2 Plasmid constructs and mutants generated in this study. P*trpC* = Aspergillus nidulans *TrpC* promoter; *Hph* = hygromycin phosphotransferase gene; T*trpC* = A. nidulans *TrpC* terminator; P*α-tub* = Zymoseptoria tritici *α-tubulin* promoter; T*α-tub = Z. tritici α-tubulin* terminator; P*gpd1* = Cochliobolus heterostrophus *Gpd1* promoter; *Gen* = geneticin resistance gene; T*β-tub* = Neurospora crassa *β-tubulin* terminator; HygR = hygromycin resistance cassette; ect = ectopic; P*avr3D1*= *Z. tritici Avr3D1* promoter; P*avrStb6 *=* Z. tritici AvrStb6* promoter; mTurq2 = mTurquoise2. Download Table S2, PDF file, 0.2 MB. Copyright © 2020 Meile et al. 2020 Meile et al. This content is distributed under the terms of the Creative Commons Attribution 4.0 International license.

10.1128/mBio.02343-20.8 TABLE S3 Primers used in this study. The cloning applications are further described in Table S2. Download Table S3, PDF file, 0.2 MB. Copyright © 2020 Meile et al. 2020 Meile et al. This content is distributed under the terms of the Creative Commons Attribution 4.0 International license.

10.1128/mBio.02343-20.9 DATA SET S1 Raw data and *mTurquoise2* sequence. (Sheet 1) Raw data of RT-qPCR (Fig. 1D; see also Fig. S1B). Crossing point (Cp) and relative expression values of *Hph* inserted ectopically compared to the same gene inserted in *Avr3D1* (*in locus*) in strain 3D1 of Zymoseptoria tritici grown under axenic conditions (YSB liquid medium). Cp and relative expression of the effector gene *Mycgr3G76589* inserted ectopically compared to the endogenous gene (*in locus*) in strain 3D7 under axenic conditions (YPD liquid medium) are also shown. (Sheet 2) Raw data of RT-qPCR (Fig. 6B and C; see also Fig. S4D). *eGFP* transcript levels during axenic growth in rich medium and during plant colonization at 11 days postinfection (dpi). The included transgenic lines of *Z. tritici* were as follows: *KMT6* wild-type (wt) and knockout (*Δkmt6* mutant), both harboring the *eGFP* gene under the control of a constitutive promoter in the *Avr3D1* locus, and a line harboring an ectopic copy of the *eGFP* cassette. Transcript levels of *AvrStb6*, *Mycgr3G76589*, and *Zt09_7_00577* during axenic growth and during plant colonization in *Z. tritici* lines with and without *KMT6* are also shown. Expression levels were normalized to the line with the wild-type *KMT6* gene during axenic growth. *Actin* was used as the reference gene for qRT-PCR. (Sheet 3) Raw data of ChIP-qPCR under axenic conditions (Fig. 4). Data represent enrichment values of H3K27me3 and H3K9me3 (measured by the percent input method). The adjusted Cp values were calculated to correct for the dilution of the input samples. (Sheet 4) Raw data of ChIP-qPCR under *in planta* and axenic conditions (Fig. 5). Data represent relative enrichment analysis of levels of histone H3 lysine 27 trimethylation and histone H3 lysine 9 trimethylation (H3K27me3 and H3K9me3, respectively) during axenic growth and during infection at the beginning of the necrotrophic phase. (Sheet 5) Raw data of ChIP-qPCR under axenic conditions (Fig. S3). Data represent enrichment values of H3K4me2, H3K27me3, and H3K9me3 (measured by the percent input method). The adjusted Cp values were calculated to correct for the dilution of the input samples. (Sheet 6) Codon-optimized sequence of *mTurquoise2*. Download Data Set S1, XLSX file, 0.04 MB. Copyright © 2020 Meile et al. 2020 Meile et al. This content is distributed under the terms of the Creative Commons Attribution 4.0 International license.

### Hygromycin growth assays.

*Z. tritici* blastospores were grown for 5 to 7 days in YSB medium. The spore suspension was filtered through cheese cloth and centrifuged (3,273 × *g*, 15 min, 4°C). Spores were resuspended in water, and the spore concentration was determined using Kova Glasstic counting chambers (Hycor Biomedical, Inc., Garden Grove, CA, USA). The concentration was adjusted to 10^6^ spores/ml, and 2.5 to 5 μl of the reaction mixture was placed on YMS agar with and without hygromycin B (100 μg/ml). Hygromycin sensitivity was assessed after 6 days of growth at 18°C.

### Infection assays.

Wheat seedlings (Triticum aestivum, cultivar Runal) were infected with *Z. tritici* blastospores as previously described (51), except for the experiments involving Δ*kmt6* mutant lines, for which blastospores were grown on YPD agar at 18°C for 3 to 5 days, washed off the agar surface with water by scraping with a pipette tip to create a spore suspension, and filtered through a 100-μm-pore-size nylon mesh. The spore concentration was adjusted to 10^6^ spores/ml (unless differently stated) in 0.1% (vol/vol) Tween 20. For symptom quantification, automated image analysis of second and third leaves was performed (87). Data were analyzed using RStudio v.1.2.5033. Kolmogorov-Smirnov tests were used with the ‘Matching’ package in RStudio for determinations of statistical significance.

### RNA isolation and quantitative reverse transcription-PCR.

Wheat leaves were harvested to obtain RNA from infected leaf tissue as described previously (51) and at least two leaves were pooled for each biological replicate. To obtain RNA from axenically grown tissue, fungal blastospores were grown on YPD agar and harvested as described above for the preparation of infection inoculum (in the case of experiments involving the Δ*kmt6* mutant) or they were grown in liquid YSB medium at 18°C for 4 to 6 days (all other experiments). In both cases, tissue was harvested by centrifugation at 4°C and flash-frozen in N_2_. RNA isolation was performed as described previously (51), and cDNA was synthesized with a RevertAid first-strand cDNA synthesis kit (Thermo Fisher Scientific, Waltham, MA, USA) following the manufacturer’s instructions and using oligo(dT)_18_ primers and up to 1,000 ng RNA per reaction. Quantitative reverse transcription-PCR (qRT-PCR) was performed on a LightCycler 480 instrument (Roche Diagnostics International AG, Rotkreuz, ZG, Switzerland). Each 10-μl reaction mixture consisted of a 250 nM concentration of each primer, template cDNA generated from up to 25 ng of RNA, and 1× HOT FIREPol EvaGreen qPCR Mix Plus master mix (Solis BioDyne, Tartu, Estonia). The amplification reactions were performed with at least three technical replicates. Primers used for qRT-PCR are listed in Table S3. Primer efficiency was determined using 5-fold serial dilutions of genomic DNA or cDNA (in the case of *AvrStb6* primers), and the data were used for efficiency-corrected calculations of the relative levels of expression (88), with *Actin* (*Mycgr3G105948*) used as the reference gene, if not stated otherwise. The expression levels of ectopic copies of the *Mycgr3G76589* gene were calculated by subtracting the value measured for the native gene in the wild-type strain from the total expression values measured in the ectopic insertion mutants. The relative expression means and standard errors of the means were calculated using RStudio v.1.2.1335 (89). Raw qPCR data are provided in Data Set S1.

### Histone deacetylase inhibition assay.

The *Z. tritici* lines used for the histone deacetylase inhibition assays carried the following transgenes: (i) *eGFP* under the control of the constitutive *α-tubulin* promoter inserted in the *Avr3D1* locus or ectopically and (ii) *mCherry* under the control of the same *α-tubulin* promoter in an ectopic position (85). For trichostatin A (TSA) treatments, 1 ml YSB medium containing 0.5 μg/ml TSA (Selleckchem, Houston, TX, USA) was inoculated with 10^5^ blastospores from glycerol stocks in a 12-well cell culture plate. Cultures were incubated at 18°C under gentle agitation for 3 days. For suberoylanilide hydroxamic acid (SAHA) treatments, 600 μl minimal medium (90) containing 1 mM SAHA (Cayman Chemical, Ann Arbor, MI, USA) was inoculated with 10^5^ blastospores from glycerol stocks in a 24-well cell culture plate. Cultures were incubated at 18°C under gentle agitation for 11 days. All treatments were performed with three biological replicates.

### Fluorescence microscopy.

Derepression of the *eGFP* cassette following treatment with histone deacetylase inhibitors and deletion of the *KMT6* gene were assessed with a Leica DM2500 fluorescence microscope equipped with a Leica DFC3000 G gray-scale camera (Leica Microsystems, Wetzlar, Germany) and the filter blocks L5 for GFP (480/40 nm excitation, 527/30 nm emission) and mCherry (580/20 nm excitation, 632/60 nm emission). Identical image processing techniques were applied to all images of each data set. Image processing included brightness and contrast adjustment, cropping, and application of Fire LUT to improve visualization of pixel gray values using the Fiji platform of ImageJ (91).

### Confocal laser scanning microscopy.

Confocal laser scanning microscopy was performed on an inverted Zeiss LSM 780 confocal microscope using a multitracking acquisition setup and the following detection settings: 490.33 to 534.72 nm for the eGFP channel, 623.51 to 641.26 nm for the mCherry channel, 656.01 to 681.98 nm for the chloroplast channel, and 459.95 to 490.07 nm for the mTurquoise2 channel. A diode-pumped solid-state laser (DPSSL) (561 nm) and an argon (488 nm) laser were used for track 1 (eGFP, mCherry, and chloroplast channels) and a diode laser (405 nm) was used for track 2 (mTurquiose2 channel). Axenically grown fungal material was suspended in 0.02% Tween 20 (for growth on solid medium) or directly observed in liquid medium. For *in planta* observations, plants were infected as described previously (51) and infected 2nd leaves were harvested immediately before observation. The top 3 cm of each leaf was discarded, and the adaxial side of the adjacent section of approximately 2 cm was observed in 0.02% Tween 20. Images were processed using the Fiji platform of ImageJ (91). Processing included cropping, adjusting brightness and contrast, adding scale bars, and generating maximum intensity z-projections. Three-dimensional (3D) reconstruction enabled us to differentiate between hyphae on the leaf surface and hyphae growing in the apoplastic space.

### Fixation of fungal and infected plant tissue for chromatin extraction.

Fixation of axenically grown fungal cells of *Z. tritici* strain 3D7 was performed as described previously (92) with the following modifications. A 5-day-old preculture was used to inoculate a 100-ml YMS culture with a starting optical density at 600 nm (OD_600_) of 0.225. This culture was grown for 38 h to an OD_600_ of between 0.79 and 0.85. Cells were fixed by adding formaldehyde to reach a final concentration of 0.5% and shaking for 15 min. Formaldehyde was quenched by adding glycine to reach a final concentration of 50 mM. Cells were washed with phosphate-buffered saline (PBS; Sigma-Aldrich, St. Louis, MO, USA), harvested by centrifugation (1 min, 800 × *g*), and flash-frozen in N_2_.

Infected 2nd leaves of cultivar Runal (subjected to spray inoculation as described above but with 5 × 10^6^ spores/ml) were harvested for chromatin preparations when the first necrosis symptoms appeared (10 to 11 days postinfection; see Fig. S5A in the supplemental material). The top 2 cm of the leaves was discarded, and the adjacent 8.5-cm sections were used for fixation. Leaf sections were cut in half, pooled (*n* = 45 to 60), and subjected to vacuum infiltration with 55 to 80 ml of fixation buffer modified from the buffer previously described by Chujo and Scott (7) (0.4 M sucrose, 10 mM Tris-HCl [pH 8.0], 1 mM EDTA, 1 mM phenylmethylsulfonyl fluoride [PMSF], 0.5% [wt/vol] formaldehyde, and 0.02% [vol/vol] Triton X-100) in a 250-ml beaker for 15 min with constant stirring. During fixation, the vacuum was released several times. Formaldehyde was quenched by adding glycine to reach a final concentration of 100 mM followed by vacuum infiltration performed for 5 min with constant stirring, releasing the vacuum several times during incubation. The leaf sections were washed twice with PBS and once with water and were then dried on paper towels and flash-frozen in N_2_.

10.1128/mBio.02343-20.5 FIG S5 Details on the chromatin immunoprecipitation procedure. (A) Phenotype of infected leaf samples used for *in planta* chromatin immunoprecipitation. Shown are approximately one-third of the leaves used for chromatin preparations on the day when the first necrotic symptoms appeared. Repl. = independent biological replicate consisting of 45 to 60 second leaves. (B and C) Optimization of the micrococcal nuclease digestion. Different incubation times were tested to optimize the micrococcal nuclease (MNase) reaction. The digestion time (15 min) that mainly released fragments of approximately 150 bp was selected as the optimum time. The gel also shows a control without MNase (-). Data represent MNase digestion of (B) chromatin extracted from axenically grown tissue obtained as described previously (Soyer et al., 2015, Fungal Genet Biol, 79:63–70, https://doi.org/10.1016/j.fgb.2015.03.006) and (C) chromatin extracted from infected wheat leaves. (D) Location of the primers used for the ChIP-qPCR. Relative locations of the primers with respect with the starting codon (position 1) of the investigated genes are indicated. Red arrows indicate the sequence from start to stop codons of each of the investigated genes. Green arrows indicate the positions of the primers. Download FIG S5, JPG file, 0.6 MB. Copyright © 2020 Meile et al. 2020 Meile et al. This content is distributed under the terms of the Creative Commons Attribution 4.0 International license.

### Chromatin preparations, immunoprecipitation, and ChIP-qPCR.

Chromatin extraction and immunoprecipitation were performed as described previously by Soyer et al. (92) with modifications. Frozen fungal or infected leaf tissue was ground using mortar and pestle. Between 150 and 233 mg of tissue was used for each chromatin extraction, which was performed similarly to a previously described method (92). Lysis buffer (50 mM HEPES-NaOH [pH 7.5], 20 mM NaCl, 1 mM Na-EDTA [pH 8.0], 1% [vol/vol] Triton X-100, 0.1% [wt/vol] sodium deoxycholate) supplied with proteinase inhibitors (1 μg/ml leupeptin, 1 μg/ml E-64, 0.5 μg/ml pepstatin A, 1 mM PMSF, and 2 μg/ml aprotinin) was added in a ratio of 5 μl to 1 mg of ground tissue in Eppendorf tubes. CaCl_2_ (1 M stock) was added to reach a final concentration of 2 mM. We optimized the micrococcal nuclease reaction by checking the chromatin digestion at different time points (Fig. S5B and C). On the basis of this optimization, we fragmented the chromatin with micrococcal nuclease (M0247S; New England Biolabs, Ipswich, MA, USA) at a concentration of 10 gel units/μl for 15 min at 37°C. Tubes were subjected to vortex mixing several times during incubation. The reaction was stopped by placing the tubes on ice and adding EGTA and EDTA (final concentration, 4 mM each). Additional NaCl (stock solution, 5 M) was added to reach a final concentration of 130 mM, and SDS (stock solution, 10% [wt/vol]) was added to reach a final concentration of 0.1%. Samples were incubated on ice for 5 min, subjected to vortex mixing several times during incubation, and subsequently cleared by centrifugation (4°C, 5 min, 1,500 × *g*). For axenically grown tissue, clearing was repeated 3 times (4°C, 5 min, 4,000 × *g*). Immunoprecipitation and de-cross-linking were performed as described previously (92) using 8.75 μg anti-histone H3K27me3 (catalog no. 39155; Active Motif, Carlsbad, CA, USA) or anti-histone H3K9me3 (Active Motif catalog no. 39161) or anti-histone H3K4me2 (EMD Millipore Corp., Billerica, MA, USA, 07-030) antibodies and 52.5 μl Dynabeads protein A (Thermo Fisher Scientific) per ml chromatin.

qPCR was carried out on a LightCycler 480 instrument (Roche) in technical duplicate using HOT FIREPol EvaGreen qPCR Mix Plus master mix (Solis BioDyne, Tartu, Estonia) and the primers shown in Table S3 (see also Fig. S5D). Three TE sequences that were unique in the 3D7 genome were selected as positive controls. The relative levels of enrichment of each target gene compared to the *TFC1* reference gene were calculated using the following equation: enrichment = eff.TFC1^Cp.TFC1^/eff.Target^Cp.Target^, where eff.TFC1 and eff.Target are the primer efficiencies for *TFC1* and the target gene, respectively, and Cp.TFC1 and Cp.Target the crossing points of *TFC1* and the target gene, respectively. The crossing point values were determined using LightCycler 480 software. Percent input (ratio of immunoprecipitated DNA to chromatin before immunoprecipitation, input) was also calculated for *in vitro* immunoprecipitations. Raw data are provided in Data Set S1.
