# Supplementary figures and images for: Chromatin Dynamics Contribute to the Spatiotemporal Expression Pattern of Virulence Genes in a Fungal Plant Pathogen
Source: mBio. 2020 Oct 6;11(5):e02343-20. doi: 10.1128/mBio.02343-20 (PMC7542367; doi:10.1128/mBio.02343-20)

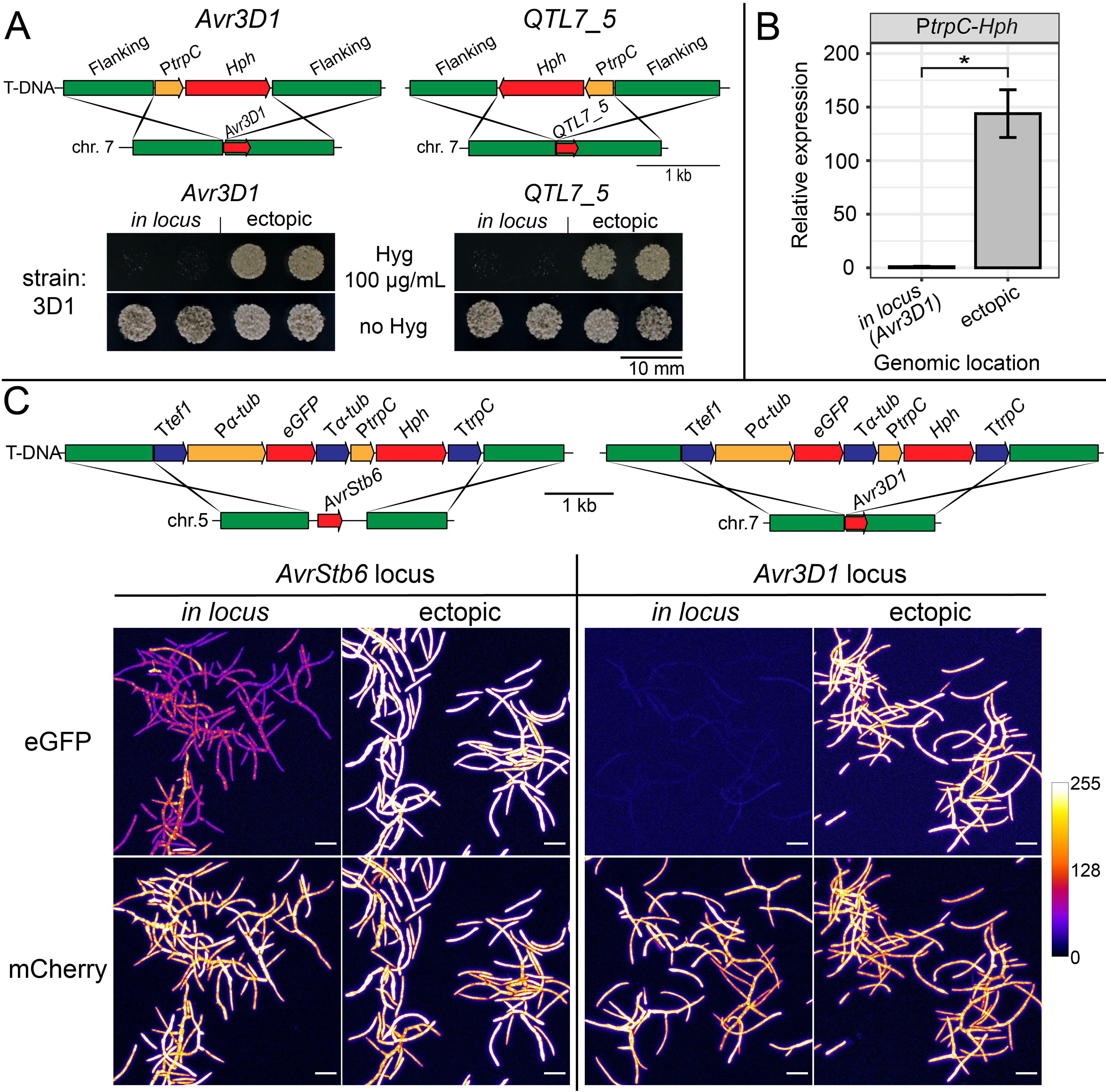

Supplement: FIG S1 [file mBio.02343-20-sf001.jpg]

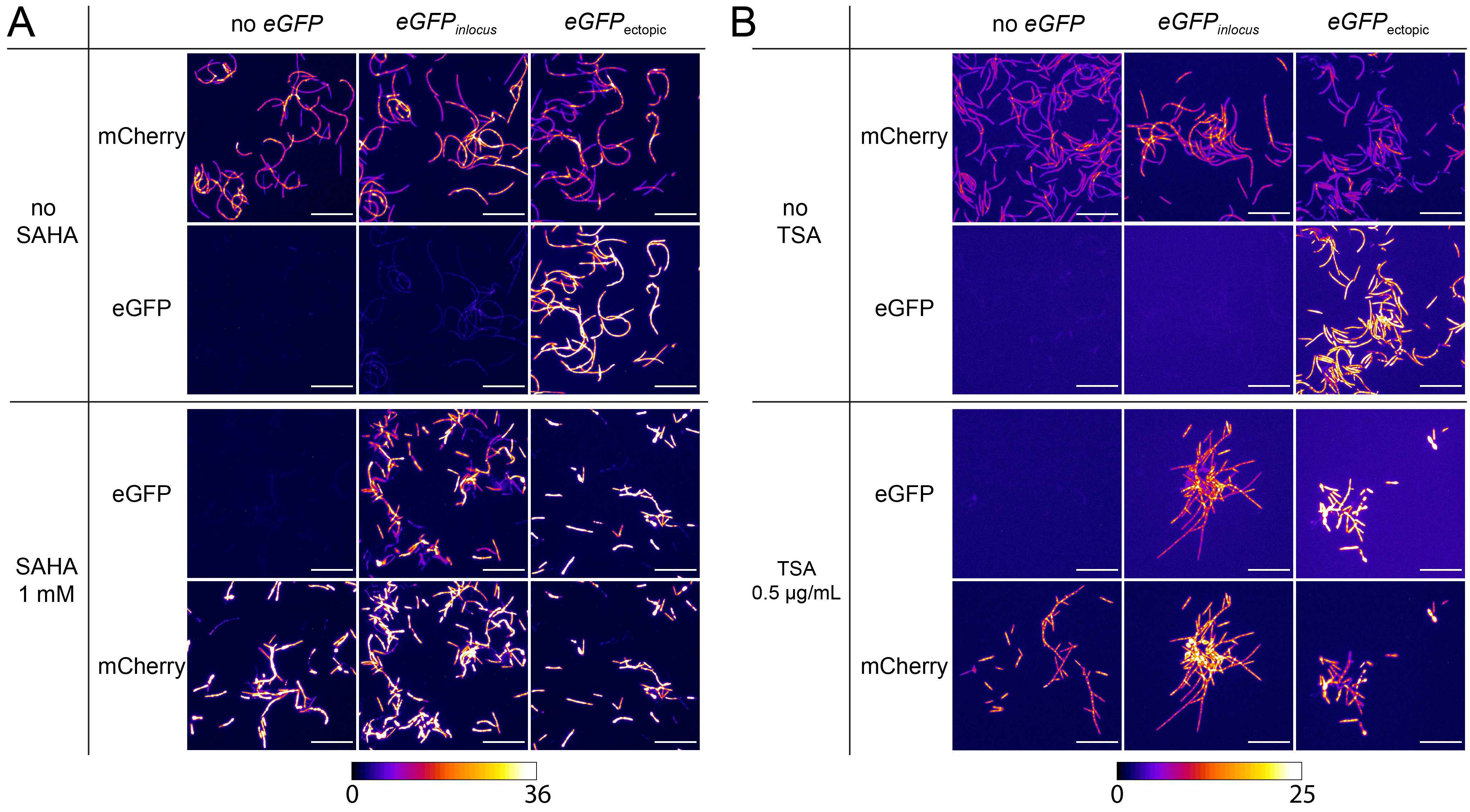

Supplement: FIG S2 [file mBio.02343-20-sf002.jpg]

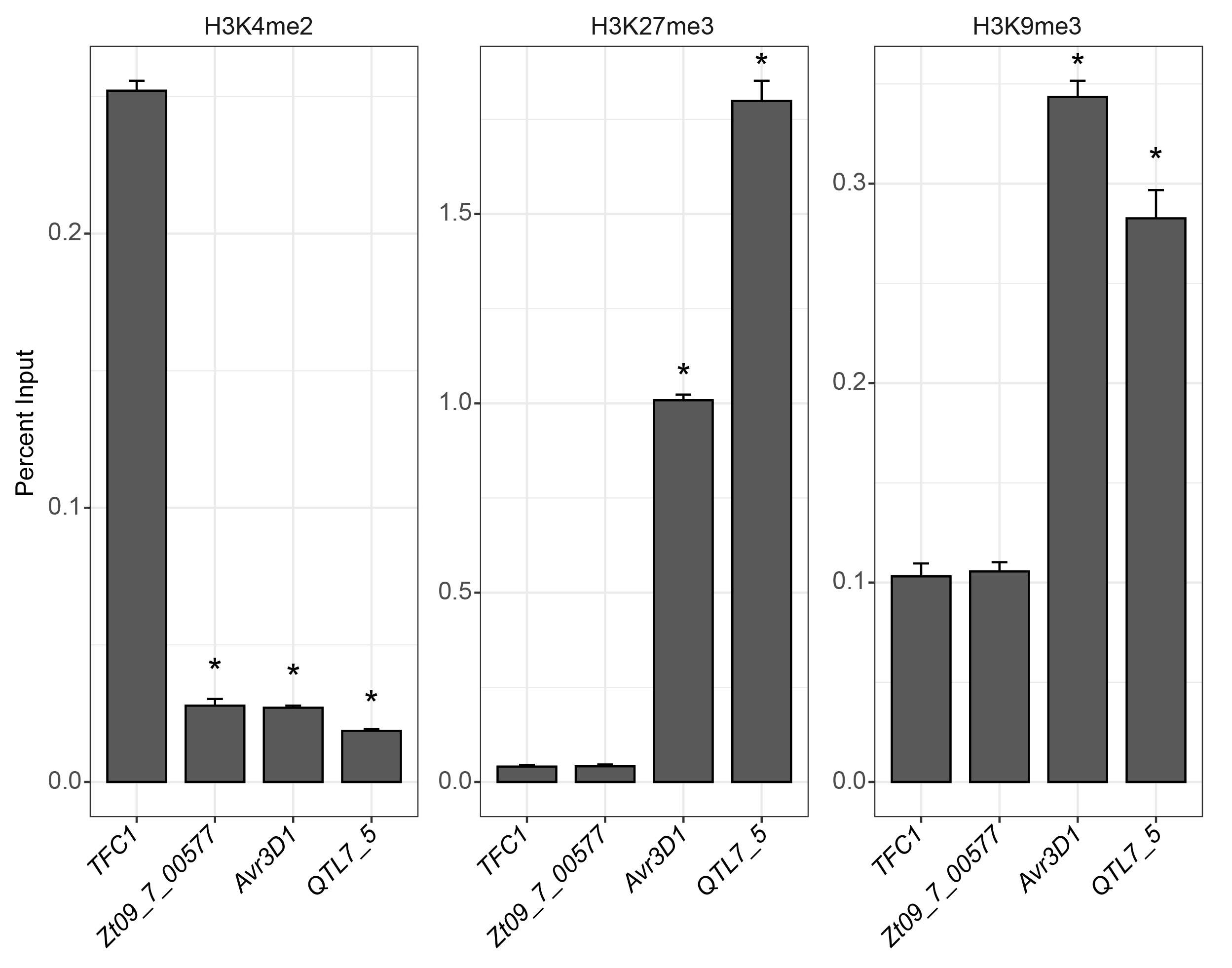

Supplement: FIG S3 [file mBio.02343-20-sf003.jpg]

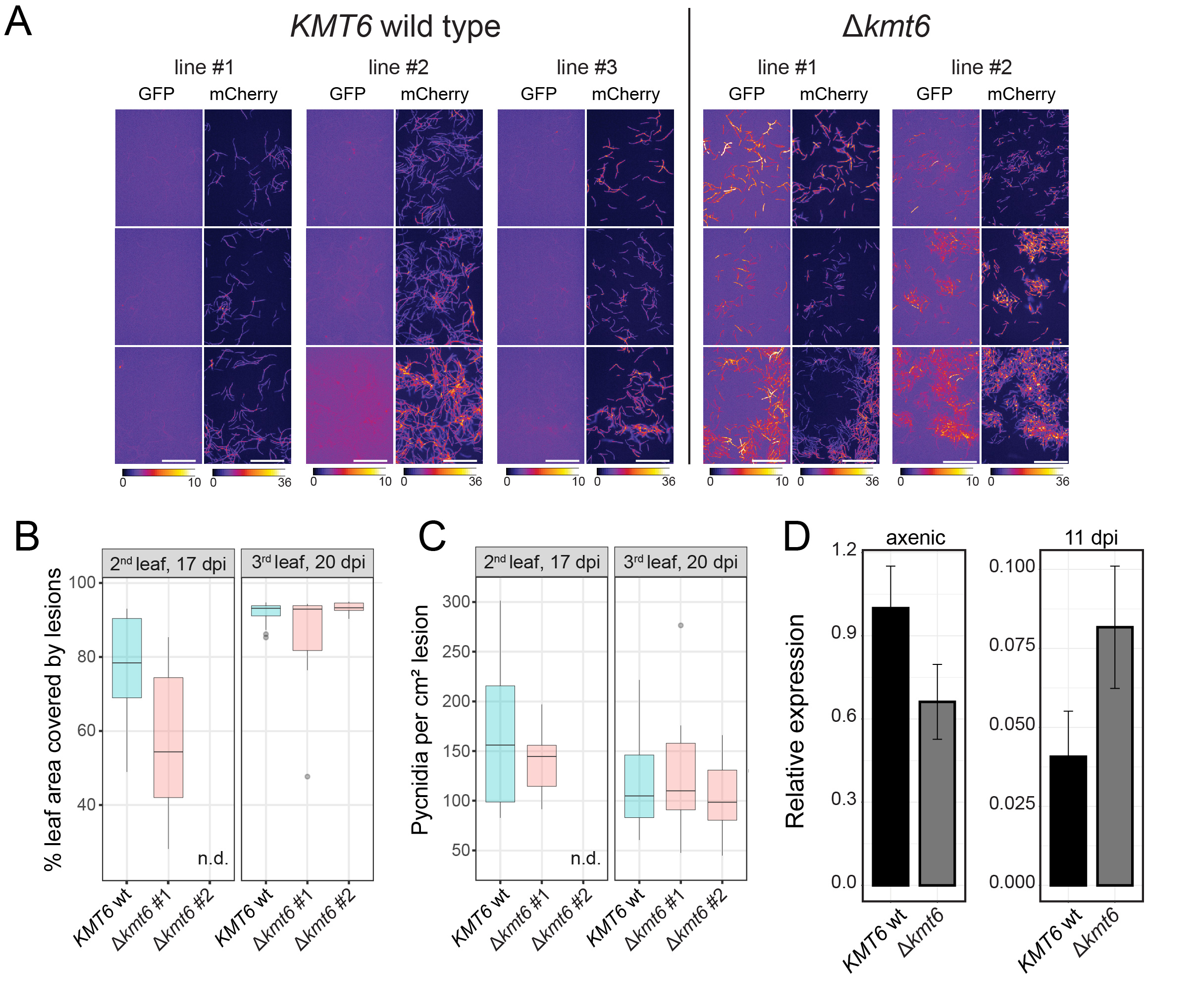

Supplement: FIG S4 [file mBio.02343-20-sf004.jpg]

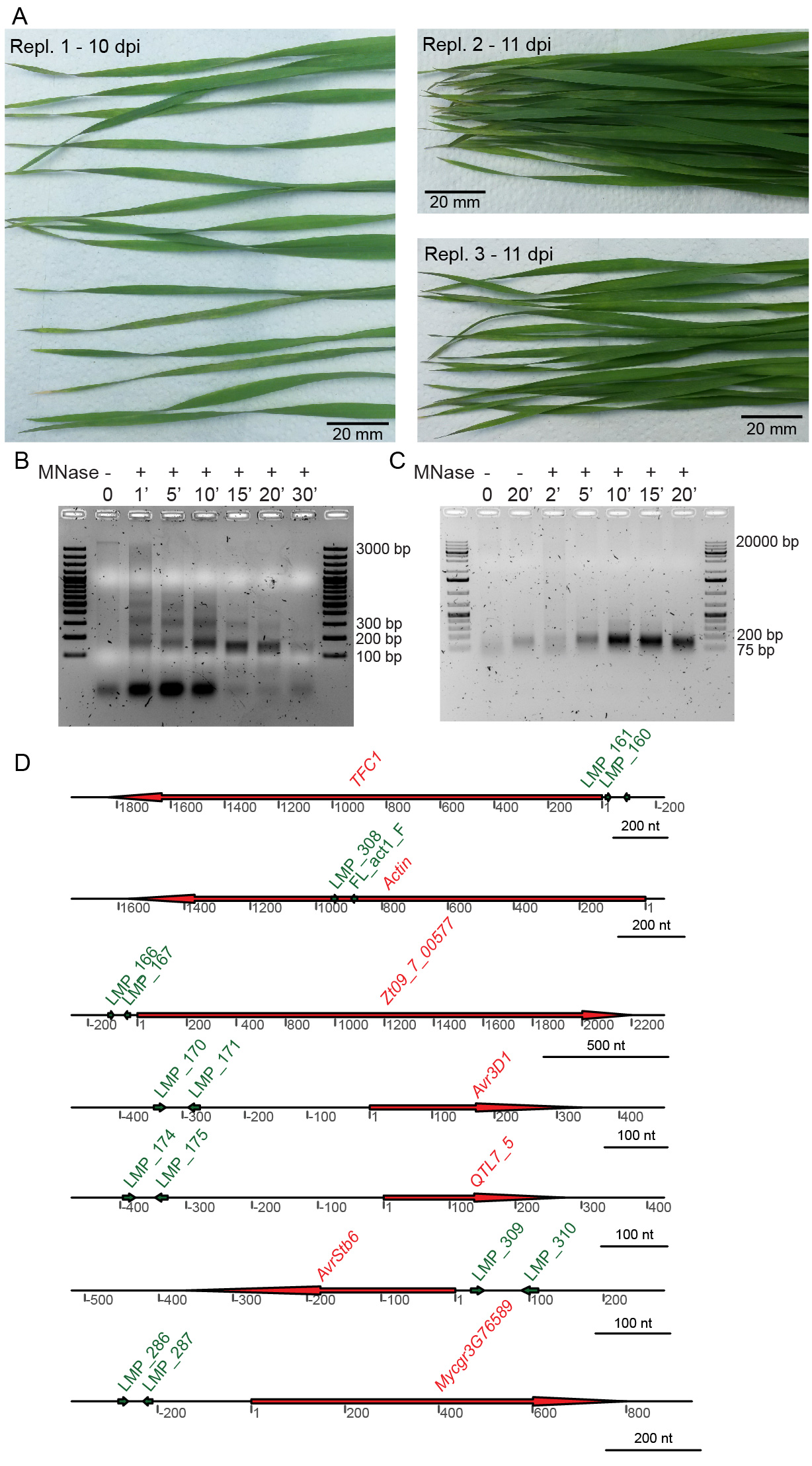

Supplement: FIG S5 [file mBio.02343-20-sf005.jpg]
