# Supplementary material for: Chromatin Dynamics Contribute to the Spatiotemporal Expression Pattern of Virulence Genes in a Fungal Plant Pathogen
Source: mBio. 2020 Oct 6;11(5):e02343-20. doi: 10.1128/mBio.02343-20 (PMC7542367; doi:10.1128/mBio.02343-20)
Supplement: TABLE S1 [file mBio.02343-20-st001.pdf]

| Gene | Gene annotations in IPO323[1] | Gene annotation in 3D7 | Genomic location in IPO323 | Genomic location in 3D7 | Distance to the closest TE upstream of the gene in 3D7 genome[4] (bp) | Chromatin context under axenic conditions in IPO323 | |
|---|---|---|---|---|---|---|---|
| | | | | | | Associated to H3K27me3[3] | Associated to H3K9me3[3] |
| *Actin* | Act/ Zt09_10_00285/ ZtritIPO323_04g02659 | 3D7.g9856 | 10:784746-786313 | 10:925374-926943 | 11538 | NO | NO |
| *TFC1* | Mycgr3G110539/ Zt09_8_00521/ ZtritIPO323_04g12870 | 3D7.g8677 | 8:1524134-1525968 | 8:1623435-1625269 | 65271 | NO | NO |
| *Zt09_7_00577* | *Zt09_7_00577*/ ZtritIPO323_04g11948 | 3D7.g7878 | 7:1870981-1873012 | 7:2011667-2013668 | 11734 | NO | NO |
| *Avr3D1* | Mycgr3G100846[2]/ *Zt09_7_00581*[2]/ *ZtritIPO323_04g11953*[2] | 3D7.g7883[2] | 7:1897693-1898030 | 7:2082428-2082768 | 1331 | YES | NO |
| *AvrStb6* | Not annotated | Not annotated | 5:69019-69383 | 5:34054-34418 | 1970 | n.d. | n.d. |
| *QTL7_5* | Not annotated | Not annotated | 7:1900513-1900788 | 7:2085251-2085526 | 4154 | n.d. | n.d. |
| *Mycgr3G76589* | *Mycgr3G76589*/ Zt09_10_00532[2]/ *ZtritIPO323_04g02937* | 3D7.g10118 | 10:1542687-1543493 | 10:1677039-1677840 | 4270 | YES | YES |

[1]Annotations according to Goodwin et al. (PLOS Genetics 7(6): e1002070, 2011, https://doi.org/10.1371/journal.pgen.1002070) /Grandaubert et al. (G3: Genes, Genomes, Genetics 5(7):1323-1333, 2015, https://doi.org/10.1534/g3.115.017731) /ZtRRes v3.0

[2] Different start codon and reading frame than manually curated annotation (this work; Meile et al. New Phytol, 219:1048-1061, 2018, doi:10.1111/nph.151802018)

[3] According to Möller et al. (PLOS Genetics 15(4): e1008093, 2019, https://doi.org/10.1371/journal.pgen.1008093)

[4] Transposable element (TE) annotation according to Plissonneau et al. (mBio 7(5):e1231-16, 2016, https//doi.org/10.1128/mBio.01231-16)
