# Supplementary material for: Chromatin Dynamics Contribute to the Spatiotemporal Expression Pattern of Virulence Genes in a Fungal Plant Pathogen
Source: mBio. 2020 Oct 6;11(5):e02343-20. doi: 10.1128/mBio.02343-20 (PMC7542367; doi:10.1128/mBio.02343-20)
Supplement: TABLE S2 [file mBio.02343-20-st002.pdf]

Plasmid	Description	Backbone	Linearization	Inserts	Description insert	Primer pair	Template	Application	Resulting mutants	Number of independent transformants analysed (Figure)
pLM1	pES1-T*tef*-P*trpC*-*Hph*-T*trpc*	pES1	KpnI, SbfI	A	P*trpC-Hph*	LMP_25/26	pES6	Template for further cloning	-	-
				B	T*tef*	LMP_148/149	pFC332			
				C	T*trpC*	LMP_150/151	pES1			
pLM2	pES1-T*tef*-P*α-tub*-*eGFP*-T*α-tub*-P*trpC*-*Hph*-T*trpc*	pLM1	SnaBI	A	P*α-tub*-*eGFP*-T*α-tub*	LMP_152/153	pCZtGFP	Template for further cloning	-	-
pLM2-Flanking$_{DOWN_AvrStb6}$	pES1-T*tef*-P*α-tub*-*eGFP*-T*α-tub*-P*trpC*-*Hph*-T*trpc*- Flanking$_{DOWN}$	pLM2	SbfI	A	Flanking$_{DOWN}$	LMP_183/185	3D7 gDNA	Template for further cloning	-	-
pLM2-Δ*AvrStb6*	pES1- Flanking$_{UP}$- T*tef*-P*α-tub*-*eGFP*-T*α-tub*-P*trpC*-*Hph*-T*trpc*-Flanking$_{DOWN}$	pLM2-Flanking$_{DOWN_AvrStb6}$	EcoRV	A	Flanking$_{UP}$	LMP_181/182	3D7 gDNA	Insertion of hygromycin resistance cassette and *eGFP* cassette in locus *AvrStb6* or ectopically	3D7-GFP-HygR$_{in\text{-}locus_AvrStb6}$,<br>3D7-GFP-HygR$_{ect_AvrStb6}$	3 (Fig 1C), 3 (Fig S1C)<br>3 (Fig 1C), 3 (Fig S1C)
pES1-Δ*Avr3D1* $_{3D7}$	pES1-Flanking$_{UP}$-P*trpC*-*Hph*-Flanking$_{DOWN}$	pES1	KpnI, PstI	A	P*trpC-Hph*	LMP_25/26	pES6	Insertion of hygromycin resistance cassette in locus *Avr3D1* or ectopically	3D7-HygR$_{in\text{-}locus_Avr3D1}$,	2 (Fig 1C)
				B	Flanking$_{UP}$	LMP_36/37	3D7 gDNA			
				C	Flanking$_{DOWN}$	LMP_38/39	3D7 gDNA		3D7-HygR$_{ect_Avr3D1}$	2 (Fig 1C)
pES1-Δ*QTL7_5* $_{3D7}$	pES1-Flanking$_{UP}$-P*trpC*-*Hph*-Flanking$_{DOWN}$	pES1	KpnI, PstI	A	P*trpC-Hph*	LMP_25/26	pES6	Insertion of hygromycin resistance cassette in locus *QTL7_5* or ectopically	3D7-HygR$_{in\text{-}locus_QTL7_5}$,	4 (Fig 1C)
				B	Flanking$_{UP}$	LMP_40/41	gDNA			
				C	Flanking$_{DOWN}$	LMP_42/43	gDNA		3D7-HygR$_{ect_ QTL7_5}$	2 (Fig 1C)
pES1-Δ*00577* $_{3D7}$	pES1-Flanking$_{UP}$-P*trpC*-*Hph*-Flanking$_{DOWN}$	pES1	KpnI, PstI	A	P*trpC-Hph*	LMP_25/26	pES6	Insertion of hygromycin resistance cassette in locus Zt09_7_*00577* or ectopically	3D7-HygR$_{in\text{-}locus_00577}$,	3 (Fig 1C)
				B	Flanking$_{UP}$	LMP_21/22	3D7 gDNA			
				C	Flanking$_{DOWN}$	LMP_23/24	3D7 gDNA		3D7-HygR$_{ect_ 00577}$	2 (Fig 1C)
pES1-Δ*Avr3D1* $_{3D1}$	pES1-Flanking$_{UP}$-P*trpC*-*Hph*-Flanking$_{DOWN}$	pES1	KpnI, PstI	A	P*trpC-Hph*	LMP_25/26	pES6	Insertion of hygromycin resistance cassette in locus *Avr3D1* or ectopically	3D1-HygR$_{in\text{-}locus_Avr3D1}$,	4 (Fig S1A), 4 (Fig S1B)
				B	Flanking$_{UP}$	LMP_36/37	3D1 gDNA			
				C	Flanking$_{DOWN}$	LMP_38/39	3D1 gDNA		3D1-HygR$_{ect_Avr3D1}$	2 (Fig S1A), 2 (Fig S1B)
pES1-Δ*QTL7_5* $_{3D1}$	pES1-Flanking$_{UP}$-P*trpC*-*Hph*-Flanking$_{DOWN}$	pES1	KpnI, PstI	A	P*trpC-Hph*	LMP_25/26	pES6	Insertion of hygromycin resistance cassette in locus *QTL7_5* or ectopically	3D1-HygR$_{in\text{-}locus_QTL7_5}$,	3 (Fig S1A)
				B	Flanking$_{UP}$	LMP_40/41	3D1 gDNA			
				C	Flanking$_{DOWN}$	LMP_42/43	3D1 gDNA		3D1-HygR$_{ect_ QTL7_5}$	2 (Fig S1A)
pLM2-Δ*Avr3D1* $_{3D7}$	pES1-Flanking$_{UP}$-T*tef*-P*α-tub*-*eGFP*-T*α-tub*-P*trpC*-*Hph*-T*trpc*-Flanking$_{DOWN}$	pES1	KpnI, SbfI	A	P*α-tub*-*eGFP*-T*α-tub*-P*trpC*-*Hph*-T*trpc*	LMP_154/155	pLM2	Insertion of *eGFP* cassette in locus *Avr3D1* or ectopically	3D7-eGFP$_{in\text{-}locus_Avr3D1}$,	2 (Fig S1 C), 1 (Fig S2), 1 (Fig 5), 1 (Fig S4B & S4C)
				B	Flanking$_{UP}$	LMP_36/156	3D7 gDNA			
				C	Flanking$_{DOWN}$	LMP_157/39	3D7 gDNA		3D7-eGFP$_{ect_Avr3D1}$	5 (Fig S1 C), 1 (Fig S2), 1 (Fig 5), 1 (Fig S4B & S4C)
pCGEN-*76589* $_{3D7\_ ect}$	pCGEN-*Mycgr3G76589*	pCGEN	KpnI	A	*Mycgr3G76589* $_{3D7}$	LMP_319/320	3D7 gDNA	Insertion of an ectopic copy of *Mycgr3G76589*	3D7-Mycgr3G76589$_{ect}$	6 (Fig 1D)
pLM2-*Pavr3D1-mTurq2*	pES1-Flanking$_{UP}$-P*avr3D1*-*mTurquoise2*-T*tef*-P*α-tub*-*eGFP*-T*α-tub*-P*trpC*-*Hph*-T*trpC*-Flanking$_{DOWN}$	pLM2	EcoRI	A	P*avr3D1* $_{3D7}$	LMP_188/189	pLM2-Δ*Avr3D1* $_{3D7}$	Insertion of *mTurq2* under the control of the native *Avr3D1* promoter and the *eGFP* cassette in locus *Avr3D1* or ectopically	3D7-P$_{Avr3D1}$mTurq2-eGFP$_{inlocus_Avr3D1}$,	2 (Fig 3)
				B	mTurquoise2	LMP_190/191	mZtTurq2 dsDNA			
				C	T*tef*-P*α-tub* $_{partial}$	LMP_192/193	pLM2-Δ*Avr3D1* $_{3D7}$		3D7-P$_{Avr3D1}$mTurq2-eGFP$_{ect_Avr3D1}$	3 (Fig 3)
pCGEN-mTurq2$_{ect}$	pCGEN-P*α-tub*- *mTurquoise2*-T*α-tub*	pCGEN	KpnI	A	P*α-tub*	LMP_140/ oJA_002	pCZGFP	Generation of a 3D7-derived strain labelled with cytosolic mTurquoise2	3D7-mTurq2$_{ect}$	
				B	*mTurquoise2*	oJA_001/003	*mZtTurq2* dsDNA			
				C	T*α-tub*	LMP_141/ oJA_004	pCZtGFP			
pLM2-*PavrStb6-His1-mCherry*	pES1-Flanking$_{UP}$-P*avrStb6*-*His1*-*mCherry*-T*tef*-P*α-tub*-*eGFP*-T*α-tub*-P*trpC*-*Hph*-T*trpC*-Flanking$_{DOWN}$	pLM2-Δ*AvrStb6*	EcoRI	A	P*avrStb6*	LMP_226/227	3D7 gDNA	Insertion of *His1-mCherry* under the control of the native *AvrStb6* promoter and the *eGFP* cassette in locus *AvrStb6* or ectopically	3D7-P$_{AvrStb6}$mCherry-eGFP$_{in\text{-}locus_AvrStb6}$	3 (Fig 2)
				B	*His1*	LMP_228/229	3D7 gDNA			
				C	*mCherry*	LMP_230/231	pCmCherry		3D7-P$_{AvrStb6}$mCherry-eGFP$_{ect_AvrStb6}$	3 (Fig 2)
				D	T*tef*-P*α-tub* $_{partial}$	LMP_232/233	pLM2-Δ*AvrStb6*			
pES1Δ*kmt6*	pES1-Flanking$_{UP}$-P*trpC*-*Hph*-Flanking$_{DOWN}$	pES1	KpnI, PstI	A	P*gpd1 Gen*-T*β-tub*	LMP_128/107	pCGEN	Generation of *Kmt6* knockout mutant	3D7-eGFP$_{in\text{-}locus_Avr3D1}$Δ*kmt6*	2 (Fig S4A & S4B & S4C), 1 (Fig 5)
				B	Flanking$_{UP}$	LMP_108/109	3D7 gDNA			
				C	Flanking$_{DOWN}$	LMP_110/111	3D7 gDNA		3D7-eGFP$_{in\text{-}locus_Avr3D1}$*KMT6* wt/ ectopic control	3 (Fig S4A)
