# Supplementary material for: Chromatin Dynamics Contribute to the Spatiotemporal Expression Pattern of Virulence Genes in a Fungal Plant Pathogen
Source: mBio. 2020 Oct 6;11(5):e02343-20. doi: 10.1128/mBio.02343-20 (PMC7542367; doi:10.1128/mBio.02343-20)
Supplement: TABLE S3 [file mBio.02343-20-st003.pdf]

Primer name	Sequence (5’-3’)	Applications	Target
LMP_25	CGGCTTTGATATTGAAGGAGC	cloning	
LMP_26	GATGGCTAGCAGATCTCTATTCC	cloning	
LMP_148	TAATTAAGATATCGAGCTCGAGGCGGACATTCGATTTATGC	cloning	
LMP_149	CTCCTTCAATATCAAAGCCGTACGTATTGGGATGAATTTTGTATGC	cloning	
LMP_150	ATAGAGATCTGCTAGCCATCGTTCATTTGTCCAAGCAGCA	cloning	
LMP_151	CAGTGCCAAGCTTGCATGCCTGCAGGCCTTCTGGGTAAACGACTCATAGG	cloning	
LMP_183	AGTCGTTTACCCAGAAGGCCGTCATCAACTTCCTCTCAACCATTCACACTCTTCTCTGGACTCCT	cloning	
LMP_185	CAGTGCCAAGCTTGCATGCCTAGTCGTTGAACTCGCCACTC	cloning	
LMP_181	TACGAATTCTTAATTAAGATGTCCGCTACCCTTATAATAACGA	cloning	
LMP_182	GGCATAAATCGAATGTCCGTGGAAAGAAGTGTGGAAGATGTG	cloning	
LMP_36	TAATTAAGATATCGAGCTCGGACTTCTTCCGACGACTTCC	cloning	
LMP_37	CTCCTTCAATATCAAAGCCGCATTGTGTCGAGGCTGGTG	cloning	
LMP_38	ATAGAGATCTGCTAGCCATCCTCCTCTTCGCCTTCTTCGG	cloning	
LMP_39	CAGTGCCAAGCTTGCATGCCAATAATCCCATCCTACCTCGCC	cloning	
LMP_40	CAGTGCCAAGCTTGCATGCCACTGCCAGATGTGTTCCTCAG	cloning	
LMP_41	ATAGAGATCTGCTAGCCATCCATTGTTGTGGATGGGTTGC	cloning	
LMP_42	CTCCTTCAATATCAAAGCCGGTTTCGCCATCTTCGCTGC	cloning	
LMP_43	TAATTAAGATATCGAGCTCGGGCTTTCGTTCAGTCAACTCG	cloning	
LMP_21	AAGATATCGAGCTCGGTACCACAACTCCACTCGCATTGAG	cloning	
LMP_22	TCAATATCAAAGCCGACTTGTACCTCTTCGTCCTCG	cloning	
LMP_23	GATCTGCTAGCCATCATGTATATCCCGTCCCTGCTG	cloning	
LMP_24	CCAAGCTTGCATGCCTGCATTAACCGCCTCATTCGTCAG	cloning	
LMP_154	GCGGACATTCGATTTATGC	cloning	
LMP_155	TTCTGGGTAAACGACTCATAGG	cloning	
LMP_156	GGCATAAATCGAATGTCCGCATTGTGTCGAGGCTGGTG	cloning	
LMP_157	TATGAGTCGTTTACCCAGAACTCCTCTTCGCCTTCTTCGG	cloning	
LMP_319	GCGCGCCGAATTCGAGCTCGGAACAACAAGGATGAACGC	cloning	
LMP_320	CCAACATGGTGGAGTGAGGGTGAATGCCATTGTCCGTG	cloning	
LMP_188	CTATGACATGATTACGAATTCGACTTCTTCCGACGACTTCC	cloning	
LMP_189	AGCTCCTCGCCCTTGGAGACCATTGTGTCGAGGCTGGTG	cloning	
LMP_190	GTCTCCAAGGGCGAGGAGC	cloning	
LMP_191	TTACTTGTAGAGCTCGTCCATGC	cloning	
LMP_140	GCCGAATTCGAGCTCGCGCCAGATGATGGCTGAGAG	cloning	
LMP_141	ACATGGTGGAGTGAGGGTACGACCGAGCTGAAGAGG	cloning	
oJA002	CCTCGCCCTTGGAGACCATGGCGATGGTGGTATGCGGATG	cloning	
oJA001	ATGGTCTCCAAGGGCGAGG	cloning	
oJA003	TTACTTGTAGAGCTCGTCC	cloning	
oJA004	GGACGAGCTCTACAAGTAAGCGACGACGGACGAGGACAGG	cloning	
LMP_226	CTATGACATGATTACGAATTCGTCCGCTACCCTTATAATAACGAG	cloning	
LMP_227	GTGACTTTCTTGGGAGGCATCTTCGTTGAATTCGAAAGGCA	cloning	
LMP_228	ATGCCTCCCAAGAAAGTCA	cloning	
LMP_229	TGCCTTCTTGGGAGTGG	cloning	
LMP_230	CCGCCACTCCCAAGAAGGCAATGGTGAGCAAGGGCGAG	cloning	
LMP_231	CGGCATAAATCGAATGTCCGTTACTTGTACAGCTCGTCCATGC	cloning	
LMP_232	CGGACATTCGATTTATGCCGTTA	cloning	
LMP_233	TGATAGCAACCCACCGAATTCTG	cloning	
LMP_128	CCCGCTTGACGACATTCC	cloning	
LMP_107	CGACGCCAGCAGTAGACAC	cloning	
LMP_108	TAATTAAGATATCGAGCTCGCCTCTTCATCTATGCCTCCT	cloning	
LMP_109	TCGGAATGTCGTCAAGCGGGAACTGCCGTGCTACTTTCTG	cloning	
LMP_110	AGTGTCTACTGCTGGCGTCGAGTAGGTAGATGTCTCTTTCGT	cloning	
LMP_111	CAGTGCCAAGCTTGCATGCCTACCACTCAAAGCCGTCCTC	cloning	
LMP_220	AGGGCATCGACTTCAAGGAG	RT-qPCR	*eGFP*
LMP_221	GTGTTCTGCTGGTAGTGGTC	RT-qPCR	
LMP_292	GTTCCGCTTCCAGTGGTTC	RT-qPCR	*Mycgr3G76589*
LMP_293	ATCCACTCCTGCTCACCAAG	RT-qPCR	
FL_act1_F	TGCCAATCTACGAGGGTTTC	RT-qPCR	*Actin*
FL_act1_R	GGATCTCCTGCTCAAAGTCG	RT-qPCR	
FL2_18344	CCAGCAAATCCTTCGATCTC	RT-qPCR	*18S*
FL2_18344	CCACTTTGACATTTCCACACC	RT-qPCR	
LMP_254	AAGGCGGGTCCTAGTTGCT	RT-qPCR	*AvrStb6*
LMP_255	AAGCTGCTGTGATGGAGAGC	RT-qPCR	
ASVP_9	CGTCTGCTGCTCCATACAAG	RT-qPCR	*Hph*
ASVP_10	CTCGATGAGCTGATGCTTTG	RT-qPCR	
LMP_349	CTTGTGTCTGCTCTTTGTCTG	RT-qPCR	*Zt09_7_00577*
LMP_350	CTGTGGTAGTGGTGGAAGTG	RT-qPCR	

FL_TFC1_F	TGCTCAGATTGTGCGAAGAC	RT-qPCR	*TFC1*
FL_TFC1_R	TCGTAGTCCGATACCATGAGG	RT-qPCR	
LMP_160	AGAGGGGTCCGTTCATCTCA	ChIP-qPCR	*TFC1*
LMP_161	GTCGAAGCAGTAGAGGCGTT	ChIP-qPCR	
FL_act1_F	TGCCAATCTACGAGGGTTTC	ChIP-qPCR	*Actin*
LMP_308	GAGGTAGTCGGTCAAATCACG	ChIP-qPCR	
LMP_166	CGAGGACGAAGAGGTACAAGTAT	ChIP-qPCR	*Zt09_7_00577*
LMP_167	AGTGGTTGTAGAAAACGAGTGAATG	ChIP-qPCR	
LMP_306	TAAAGGCATTGTCTCCGACAG	ChIP-qPCR	TE_RLG_element3
LMP_307	GGTCACATGCGATTCCCAAC	ChIP-qPCR	
LMP_300	ATTAGTGTCTCTGTCGCCGTC	ChIP-qPCR	TE_DTX_element6
LMP_301	TCGCAATCTCCCTTATTATCTCC	ChIP-qPCR	
LMP_296	GTTAATATCCTCCGTAGCCGAAT	ChIP-qPCR	TE_DTH_element10
LMP_297	CACTACTACCGCTATAACTACCCT	ChIP-qPCR	
LMP_170	AGCATTCGACGACTGTTGGT	ChIP-qPCR	*Avr3D1*
LMP_171	GGTGGCTAGCTTGGAACTGT	ChIP-qPCR	
LMP_309	AATGGATTCGGCGACAGGT	ChIP-qPCR	*AvrStb6*
LMP_310	TTAGGTCATCAACTTCCTCTCAAC	ChIP-qPCR	
LMP_174	AATTGAGCCGAGGACCAAGG	ChIP-qPCR	*QTL7_5*
LMP_175	CGATGTGGGAGGCAGATGAA	ChIP-qPCR	
LMP_286	GTCTGGAGTAGATTAGCCTCGC	ChIP-qPCR	*Mycgr3G76589*
LMP_287	CACCAACGAAAGTCACGAAAC	ChIP-qPCR	
